# Metabolic Phenotyping from Whole-Blood Responses to a Standardized Exercise Test May Discriminate for Physiological, Performance, and Illness Outcomes: A Pilot Study in Highly-Trained Cross-Country Skiers

**DOI:** 10.1186/s40798-024-00770-0

**Published:** 2024-09-18

**Authors:** Øyvind Karlsson, Andrew D. Govus, Kerry McGawley, Helen G. Hanstock

**Affiliations:** 1 Swedish Winter Sports Research Centre, Department of Health Sciences, Mid Sweden University, Studentplan 4, Östersund, 831 40 Sweden; 2 Department of Sport, Exercise, and Nutrition, La Trobe University, Melbourne, VIC Australia

**Keywords:** Athlete monitoring, Endurance athletes, Metabolic profiling, Physiological testing, Health

## Abstract

**Background:**

This study used metabolic phenotyping to explore the responses of highly-trained cross-country skiers to a standardized exercise test, which was part of the athletes’ routine testing, and determine whether metabolic phenotyping could discriminate specific physiological, performance, and illness characteristics.

**Methods:**

Twenty-three highly-trained cross-country skiers (10 women and 13 men) participated in this study. Capillary whole-blood samples were collected before (at rest) and 2.5 min after (post-exercise) a roller-ski treadmill test consisting of 5–6 × 4-min submaximal stages followed by a self-paced time trial (~ 3 min) and analyzed using mass spectrometry. Performance level was defined by International Ski Federation distance and sprint rankings. Illness data were collected prospectively for 33 weeks using the Oslo Sports Trauma Research Center Questionnaire on Health Problems. Orthogonal partial least squares-discriminant analyses (OPLS-DA) followed by enrichment analyses were used to identify metabolic phenotypes of athlete groups with specific physiological, performance, and illness characteristics.

**Results:**

Blood metabolite phenotypes were significantly different after the standardized exercise test compared to rest for metabolites involved in energy, purine, and nucleotide metabolism (all OPLS-DA *p* < 0.001). Acute changes in the metabolic phenotype from rest to post-exercise could discriminate athletes with: (1) higher vs. lower peak blood lactate concentrations; (2) superior vs. inferior performance levels in sprint skiing, and (3) ≥ 2 vs. ≤ 1 self-reported illness episodes in the 33-week study period (all *p* < 0.05). The most important metabolites contributing to the distinction of groups according to (1) post-exercise blood lactate concentrations, (2) sprint performance, and (3) illness frequency were: (1) inosine, hypoxanthine, and deoxycholic acid, (2) sorbitol, adenosine monophosphate, and 2-hydroxyleuroylcarnitine, and (3) glucose-6-phosphate, squalene, and deoxycholic acid, respectively.

**Conclusion:**

Metabolic phenotyping discriminated between athlete groups with higher vs. lower post-exercise blood lactate concentrations, superior vs. inferior sprint skiing performance, and more vs. less self-reported illnesses. While the biological relevance of the identified biomarkers requires validation in future research, metabolic phenotyping shows promise as a tool for routine monitoring of highly-trained endurance athletes.

**Supplementary Information:**

The online version contains supplementary material available at 10.1186/s40798-024-00770-0.

## Key Points

• The standardized exercise test led to alterations in metabolites primarily involved in carbohydrate metabolism and the tricarboxylic acid cycle, purine/pyrimidines, and nucleoside/nucleotides.

• Acute changes in metabolic phenotypes could differentiate athletes with higher vs. lower [La-]peak and superior vs. inferior sprint skiers, and between athletes who were susceptible vs. non-susceptible to illness.

• Metabolic phenotyping shows promise as a tool for routine monitoring of endurance athletes if cost and turnaround time is improved.

**Supplementary Information:**

The online version contains supplementary material available at 10.1186/s40798-024-00770-0.

## Background

Biomarkers contained within biological fluids, such as blood, urine, and saliva, are commonly measured to monitor athletes’ responses to training and health status [1–3]. However, current athlete monitoring approaches typically measure only a single or a limited set of biomarkers when attempting to characterize complex physiological processes associated with exercise and health [4–7]. Given that the body functions as an integrative system, reductionist approaches provide incomplete information about the effect of exercise on biomarkers of athlete performance and health [6, 8]. Thus, there is a need to explore alternative approaches that more accurately reflect the complex processes associated with training and health outcomes.

Metabolomics provides a readout of hundreds to thousands of metabolites simultaneously in a given biofluid (e.g., blood, urine, or saliva), thereby providing a comprehensive representation of the metabolic state of the organism at the time of sample collection (i.e., a metabolic phenotype) [5, 8]. Hence, a major advantage of metabolic phenotyping compared to traditional biomarker approaches is that multiple metabolic pathways can be assessed simultaneously [5, 9]. Moreover, only a small volume of biofluid (i.e., ~ 0.1 mL) is necessary for metabolic phenotyping, whereas measuring a single biomarker (such as creatine kinase, for example) can require a similar sample volume. Smaller samples make frequent sampling during exercise in a laboratory or the field possible, thereby facilitating a more complete understanding of an athlete’s metabolic response to training and their current physical state.

Several studies have used metabolomics to describe the metabolic response to exercise in athletes and demonstrate potential for the method to differentiate athletes with different physiological capacities. For example, Monnerat et al. [10] reported distinct differences in resting metabolic phenotypes and changes after a standardized exercise test in elite endurance runners grouped according to peak oxygen consumption (V̇O_2peak_; high: 76.3 ± 1.5 mL∙kg^−1^∙min^−1^ vs. low: 61.0 ± 3.5 mL∙kg^−1^∙min^−1^). Similarly, San-Millán et al. [6] reported distinct differences in resting and post-exercise metabolic phenotypes after a graded exercise test to exhaustion in World Tour professional cyclists grouped according to their blood lactate concentration ([La^−^]) when cycling at 5.0 W∙kg^−1^. Specifically, characteristics of athletes with [La^−^] below the group average of 5 mmol L^−1^ included elevated basal tyrosine, amino acid, and oxidative stress markers, as well as greater post-exercise tricarboxylic acid (TCA) cycle metabolites and greater tyrosine and branched-chain amino acid (BCAA) consumption after exercise [6]. Although previous research has successfully differentiated highly-trained athletes in activities such as running [10] and cycling [6], there is a need for replication (due to typically small sample sizes) and investigation of other athlete groups and exercise modes to determine key metabolites and pathways associated with sport specific endurance performance. To the best of the authors’ knowledge, no investigation has yet used metabolic phenotyping to explore metabolic responses to acute whole-body exercise (i.e., roller-skiing) in highly-trained cross-country (XC) skiers, nor in a sample of both highly trained female and male endurance athletes.

Metabolic phenotyping has also shown promise in differentiating athletes of different performance levels. For example, Shader et al. [11] reported significant differences in the post-exercise metabolomic phenotypes of marathon runners grouped according to their race performance and aerobic fitness, which was defined as peak oxygen consumption (V̇O_2peak_). Specifically, post-race plasma concentrations of acylcarnitines and arginine-related metabolites were higher in the slower, less aerobically fit runners, compared to their faster, more aerobically fit counterparts [11]. In addition, Cai et al. [12] were able to differentiate elite and sub-elite swimmers based on resting serum samples. The elite swimmers showed higher relative abundances of high-density lipoproteins, unsaturated fatty acids, lactic acid, and methanol, and lower relative abundances of isoleucine, 3-hydroxybutyric acid, acetoacetate, glutamine, glycine, and α-glucose [12]. However, no study to date has differentiated highly-trained XC skiers based on their metabolic phenotype at rest or after exercise. Furthermore, metabolic phenotyping has demonstrated potential in identifying metabolites associated with illness susceptibility in athletes. Cuthbertson et al. [13] reported significant differences in 10 metabolites predominantly active in the sphingomyelin and ceramide sub-pathways in the resting metabolic phenotypes of Olympic-level athletes susceptible to respiratory tract infections (RTIs) (i.e., experiencing ≥ 4 RTIs within 18 months) compared to non-susceptible athletes (i.e., experiencing ≤ 1 RTI within 18 months). The ability to identify an athlete’s risk of illness could be valuable when planning and managing elite athletes’ training, recovery, and health strategies [14].

Routine physiological laboratory testing is common in elite sports and offers a unique opportunity for the regular monitoring of metabolic phenotype, as test protocols provide highly standardized sampling conditions and exercise stimuli. In addition, if incorporated as part of athletes’ regular monitoring, interruptions to training and/or recovery routines are minimized. Thus, the aims of this pilot study were to: (1) examine the metabolic phenotypes of highly-trained endurance athletes before and after a standardized exercise test that was part of their regular physiological performance profiling; and (2) explore whether the resting metabolic phenotypes or post-exercise changes in the metabolic phenotypes can discriminate between athlete groups separated by physiological, performance, and illness characteristics.

## Materials and methods

### Participants

Twenty-three healthy, highly-trained [15] XC skiers (10 women and 13 men) were recruited via convenience sampling using the following criteria: (1) selected by the Swedish Ski Association to represent the national development XC ski team and (2) over 18 years of age at the time of inclusion. Participant characteristics are displayed in Table 1. The study was preapproved by the regional ethical review board in Umeå, Sweden (ref: 2018-46-31 M), and written informed consent was obtained from all athletes before participation.

**Table 1:** Participant characteristics

	All		Women		Men	
	Mean	SD	Mean	SD	Mean	SD
n	23		10		13	
Age (yr)	20.5	1.3	20.3	1.5	20.7	1.2
Height (cm)	176.7	6.2	171.5	2.9	180.8	4.8
Body mass (kg)	71.1	6.9	64.8	3.3	76.0	4.5
V̇O_2peak_ (L∙min^−1^)	4.77	0.77	3.96	0.24	5.40	0.26
V̇O_2peak_ (mL∙kg^−1^∙min^−1^)	66.8	5.6	61.2	1.4	71.1	3.2
%V̇O_2peak_@2mmol	76.7	4.4	78.1	3.3	75.6	4.9
%V̇O_2peak_@4mmol	86.6	3.4	87.6	2.4	85.7	3.9
FIS distance points	68.3	15.3	75.4	13.0	62.8	15.2
FIS distance rank	309	161	221	79	377	177
FIS sprint points	132.2	55.2	112.6	61.6	147.3	46.7
FIS sprint rank	398	290	216	173	537	288

*Abbreviations* FIS = International Ski Federation; V̇O_2peak_ = peak oxygen consumption; %V̇O_2peak_@2mmol = % of V̇O_2peak_ at a blood lactate concentration of 2 mmol∙L^− 1^; %V̇O_2peak_@4mmol = % of V̇O_2peak_ at a blood lactate concentration of 4 mmol∙L^−1^

### Experimental Overview

**Fig. 1:**
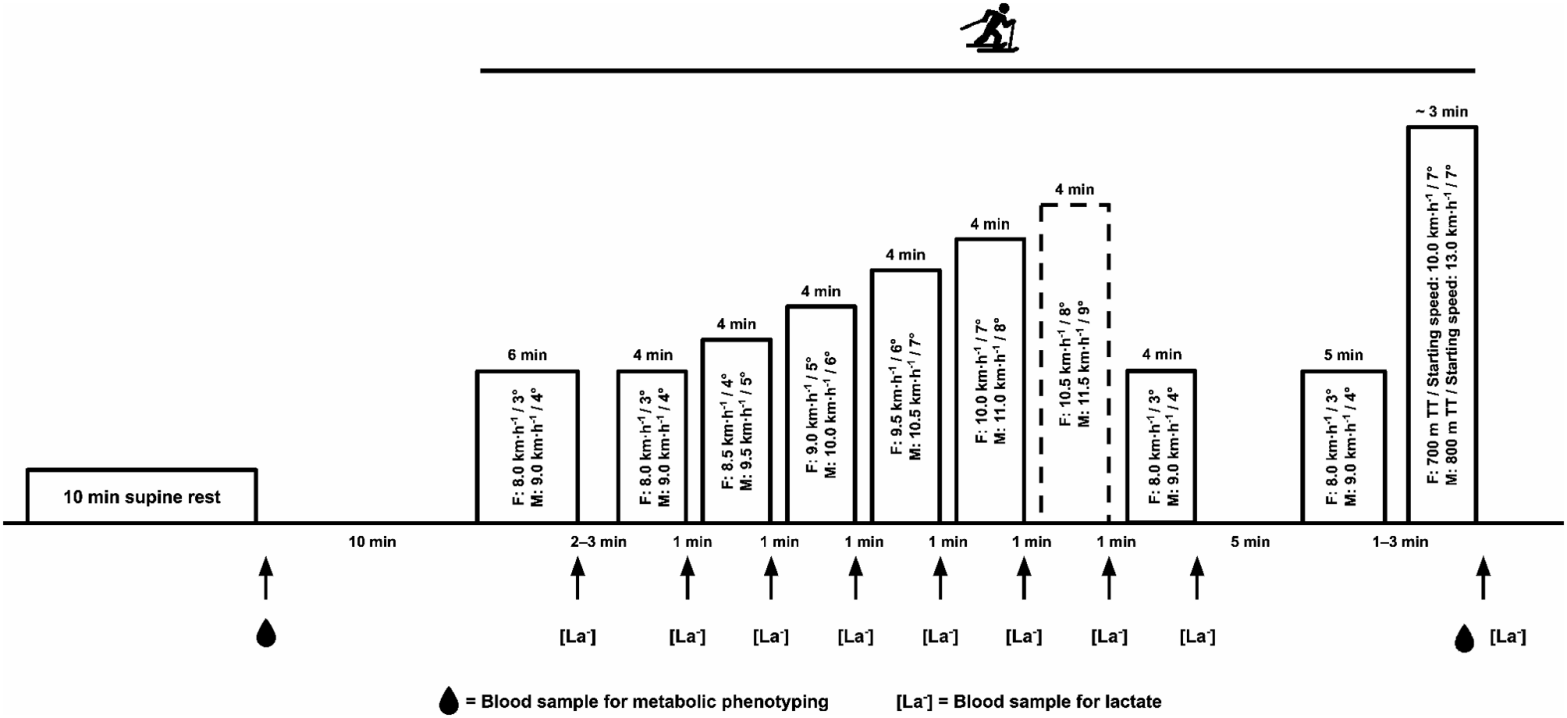
Schematic of the standardized exercise test displaying the duration (min), speed (km∙h^− 1^) and inclination (°) for each stage for female and male skiers, respectively. Capillary whole-blood samples were collected before (at rest) and after (post-exercise) the test for later metabolomic analysis. Capillary whole-blood samples were also collected after each submaximal stage and after the time trial to determine speed and percentage of peak maximal oxygen consumption corresponding to blood lactate concentrations of 2 and 4 mmol·L^− 1^and the peak blood lactate concentration, respectively. F: female skiers. M: male skiers. TT: time trial. Solid lines: performed by all athletes. Dotted lines: completed depending on fitness/capacity

The athletes completed a standardized exercise test as part of their annual pre-season physiological monitoring routine (Fig. 1). Athletes were instructed not to exercise the day before the test and to avoid prior physical activity on the day of the test. Capillary whole-blood samples (minimum of 45 µL) were collected before (rest) and after (post-exercise) the standardized exercise test for subsequent metabolic phenotyping. Previous research has demonstrated the efficacy of capillary whole-blood samples in revealing changes in the metabolic phenotype of highly-trained endurance athletes (6). Capillary whole-blood samples (20 µL) were also collected to determine the speeds and percentages of V̇O_2peak_ (%V̇O_2peak_) corresponding to [La^−^] values of 2 and 4 mmol·L^−1^ and the peak [La^−^] ([La^−^]_peak_). In addition, information regarding illness episodes was collected weekly for 33 weeks as part of the athletes’ regular monitoring [16], from a pre-season training camp in September until the end of the XC ski season in April the following calendar year, using the Oslo Sports Trauma Research Center Questionnaire on Health Problems (OSTRC-H2) [17]. Finally, on-snow sprint and distance skiing performance were determined using International Ski Federation (FIS) points and rankings [18], which were retrieved from fis.com for the timepoint corresponding to completion of the standardized exercise test.

### Standardized Exercise Test

The standardized exercise test involved roller skiing on a motorized treadmill (belt dimensions, 3.3 × 2.5 m; Rodby Innovation AB, Vänge, Sweden) according to Jones et al. [19]. Briefly, after a 6-min warm-up, the athletes performed 5–6 × 4-min submaximal stages separated by 1-min passive rest intervals. The submaximal part of the test was terminated after the stage at which the respiratory exchange ratio exceeded 1.00, ventilatory equivalent of oxygen exceeded 30, and heart rate exceeded 90% of self-reported maximal heart rate [19]. Thus, the total number of completed stages depended on the athlete’s fitness (all athletes performed at least 5 stages). After a 4-min recovery at the same speed and inclination as the warm-up, and 5 min of passive recovery, the skiers performed a 5-min re-warm-up before commencing the self-paced time trial (TT). The TT was carried out on a 7° inclination over 700 m for the women (starting speed, 10 km·h^− 1^) and 800 m for the men (starting speed, 13 km·h^− 1^). The TT lasted 3:09–3:34 min: s and the total duration of the standardized exercise test was between 45 and 60 min.

V̇O_2_ and heart rate were monitored continuously throughout the test using an AMIS 2001 metabolic system (model C; Innovision A/S, Odense, Denmark) and a watch and chest strap (Polar V800; Polar Electro Oy, Kempele, Finland), respectively. Capillary blood samples for determining speeds and %V̇O_2peak_ corresponding to [La^−^] values of 2 and 4 mmol·L^−1^ and [La^−^]_peak_ were analyzed immediately after collection from the fingertip (Biosen S-Line, EKF diagnostic GmbH, Magdeburg, Germany) after the completion of each submaximal stage and 2 min after the TT. The highest consecutive 30-s V̇O_2_ values measured during the TT were reported as the athlete’s V̇O_2peak_ [20]. Speed and %V̇O_2peak_ corresponding to [La^−^] values of 2 and 4 mmol·L^− 1^ were calculated from the individual linear relationships between speed and [La^−^] and V̇O_2_ and [La^−^], respectively [19].

### Blood Sample Collection and Handling for Metabolic Phenotyping

The same experienced laboratory technician collected all the whole blood capillary samples for subsequent metabolic phenotyping. Resting samples were collected after 10 min of supine rest, while post-exercise samples were collected 2.5 min after the completion of the TT [21], immediately after and from the same finger-prick as the [La^−^]_peak_-samples. Samples were obtained from the sterilized tip of the second, third, or fourth finger using a 21G safety lancet (Sarstedt, Nümbrecht, Germany). The initial blood droplet was blotted with a clean gauze, after which the sample was collected into a 100 𝜇L Microvette^®^ 100 K3 EDTA tube (Sarstedt, Nümbrecht, Germany). Two 40 𝜇L aliquots were then transferred to Eppendorf tubes and stored at − 80 °C within 5 min for later metabolic phenotyping.

### Metabolic Phenotyping

Metabolic phenotyping was performed at the Swedish Metabolomics Center in Umeå, Sweden, using both targeted and untargeted approaches according to A et al. [22]. Briefly, the targeted approach involved searching for a pre-defined set of metabolites (*n* = 26, see Table 1 in Electronic Supplementary Material 1) from the TCA cycle, glycolysis, and amino acid metabolism within the data obtained with gas chromatography-mass spectrometry (GC-MS). Absolute quantifications were achieved by matching accurate masses and retention times with internal standards. The untargeted approach aimed to achieve relative quantification of all detectable metabolites in the blood sample using both GC-MS and liquid chromatography-mass spectrometry (LC-MS). To control for possible instrumental drift, sample run order was randomized and concentrations of identified metabolites were adjusted according to internal standards. For a detailed description of the metabolic phenotyping procedure, readers are referred to Electronic Supplementary Material 1.

### Athlete Groupings

Within each sex, mean splits were used to categorize athletes into high and low groups for the following variables: time to complete the TT, [La^−^]_peak_, absolute and relative V̇O_2peak_ and speed and %V̇O_2peak_ at [La^−^] values of 2 and 4 mmol∙L^−1^. Subsequently, the high and low female and male athletes were consolidated into mixed-sex high and low groups for each variable to increase statistical power. These consolidated groups were then subjected to further analyses. Similarly, within each sex, a median split was used to group athletes according to superior and inferior on-snow sprint and distance skiing performance before consolidating the athletes into mixed-sex high and low groups for further analyses. Illness-susceptible (S) athletes were defined as reporting ≥ 2 illness episodes in the 33 weeks after the standardized exercise test, while non-susceptible (NS) athletes were defined as reporting ≤ 1 illness episode during the same time period, with both groups comprising both sexes. Group characteristics are presented in Table 2.

**Table 2:** Group characteristics for significant orthogonal partial least squares discriminant analysis models

	Post-exercise lactate concentration				Sprint performance				Illness susceptibility			
	High		Low		High		Low		Susceptible		Non-susceptible	
	Mean	SD	Mean	SD	Mean	SD	Mean	SD	Mean	SD	Mean	SD
Women/men (n)	6/7		3/7		5/7		5/6		7/7		3/5	
Age (yr)	20.9	1.5	20.2	1.2	21.1	1.1	20.1	1.4	20.5	1.5	20.8	1.0
Height (cm)	175.5	5.8	178.3	6.6	176.7	6.8	176.8	5.8	176.0	5.2	178.1	7.9
Body mass (kg)	70.2	6.7	72.3	7.4	73.0	7.9	69.1	5.3	69.3	5.1	74.5	8.9
V̇O_2peak_ (L∙min^−1^)	4.67	0.75	4.90	0.81	4.87	0.76	4.67	0.80	4.65	0.75	5.00	0.79
V̇O_2peak_ (mL∙kg^−1^∙min^−1^)	66.3	6.0	67.3	5.4	66.4	4.5	67.2	6.9	66.7	6.3	66.9	4.5
%V̇O_2peak_@2mmol	75.6	4.7	78.1	3.6	76.8	4.0	76.5	4.9	76.9	4.4	76.2	4.4
%V̇O_2peak_@4mmol	85.8	3.4	87.7	3.1	86.6	3.1	86.5	3.7	86.6	3.3	86.4	3.6
[La^−^]_peak_	16.0	1.5	12.3	1.3	14.3	2.1	14.5	2.7	14.1	2.3	15.0	2.4
FIS distance points	71.37	15.65	64.33	14.73	66.59	11.50	70.19	19.09	71.46	16.25	62.41	12.25
FIS distance rank	336	192	275	108	314	180	305	146	309	167	310	161
FIS sprint points	126.26	64.85	139.99	41.58	93.87	42.91	174.07	31.51	145.57	51.69	107.22	56.08
FIS sprint rank	373	329	430	244	231	195	580	271	433	309	331	255

Abbreviations FIS = International Ski Federation; [La^−^]_peak_ = peak lactate concentration post-exercise, V̇O_2peak_ = peak oxygen consumption; %V̇O_2peak_@2mmol = % of V̇O_2peak_ at a blood lactate concentration of 2 mmol∙L^− 1^; %V̇O_2peak_@4mmol = % of V̇O_2peak_ at a blood lactate concentration of 4 mmol∙L^−1^

### Statistical Analyses

Data are expressed as mean ± standard deviation (SD) unless stated otherwise. Data from the targeted and untargeted metabolic phenotyping approaches were combined into a single dataset before analysis. Duplicate metabolites detected by more than one approach were initially retained to identify the most suitable approach for separating athlete groups. To quantify changes in the athletes’ metabolic phenotype from rest to post-exercise, the log_2_ fold-change in concentration (targeted approach) or relative abundance (untargeted approach) was calculated for each detected metabolite. To illustrate acute changes in the metabolic phenotype in response to the standardized exercise test, the top 50 *t*-test (rest vs. post-exercise) significant metabolites (i.e., smallest *p* values) were hierarchically clustered (distance measure: Euclidian distance; clustering method: Ward) and presented in a heatmap using MetaboAnalyst 5.0 [23].

To analyze group differences, unsupervised principal component analyses (PCA) and supervised orthogonal partial least squares discriminant analyses (OPLS-DA) were carried out using the autofit function in SIMCA 16.0 (Sartorius AG, Umeå, Sweden). Separate PCA and OPLS-DA models for each grouping variable were created with the resting and log_2_ fold-change (from rest to post-exercise) data. Before analysis the resting data were log_10_ transformed and Pareto scaled, while the log_2_ fold-change data were Pareto scaled only. Model fit and predictive ability were assessed using R^2^Y (i.e., the cumulative fraction of the variation of the response explained by the model up to the specified component) and Q^2^ (i.e., the cumulative fraction of the variation of the response predicted by the model, up to the specified component, according to leave-one-out cross-validation), respectively [24]. R^2^Y and Q^2^ should be > 0.5 and the difference between R^2^Y and Q^2^ should not be > 0.3 for well-modeled data [24]. Statistical significance of OPLS-DA models was assessed using cross-validation analysis of variance (CV-ANOVA) [24]. To evaluate the importance of the metabolites to group distinction, variable influence on projection (VIP) analyses were executed [24]. Initial OPLS-DA models that did not reach statistical significance were refined by excluding metabolites with VIPs < 1 in the initial model. All non-significant initial and refined OPLS-DA models are summarized in Electronic Supplementary Material 2 and 3, respectively.

Statistically significant OPLS-DA models were followed up by metabolite quantitative enrichment analyses to evaluate the biological significance of the metabolic changes. If duplicates existed (i.e., metabolites detected with more than one analysis approach, for example, with both GC-MS and LC-MS), the one with the highest VIP score was retained in the enrichment analysis. Metabolite enrichment analyses were carried out in MetaboAnalyst 5.0 [23]. Adjustments for false discovery rate (FDR) of *p*-values in the enrichment analysis was carried using the Benjamini-Hochberg method.

## Results

The targeted approach successfully detected and quantified 24 out of the 26 pre-defined metabolites (see Table 1 in Electronic Supplementary Material 1). The untargeted approach successfully identified, and relative quantifications were obtained for, a total of 306 unique metabolites across all samples: 28 using GC-MS, 262 using LC-MS, and 16 using both GC-MS and LC-MS. A list of the identified metabolites with their measured mass retention, time, score, and relative standard deviations are presented in Electronic Supplementary Material 4.

### Acute Changes in the Metabolic Phenotype in Response to Exercise

**Fig. 2:**
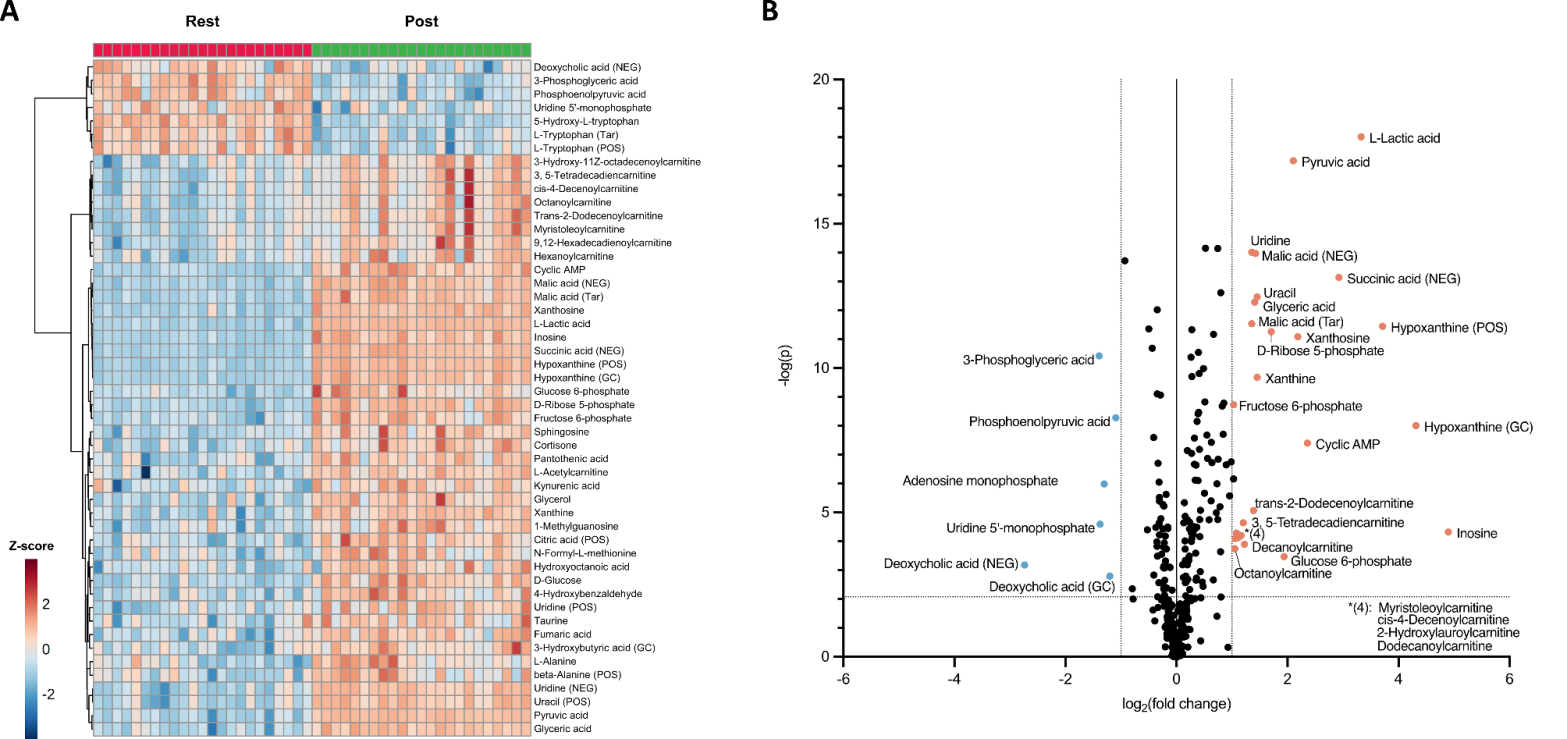
**A**) Heatmap with dendrogram depicting the hierarchical clustering analysis of the top 50 *t*-test significant Z-score normalized metabolites. **B**) Volcano plot depicting -log_10_(*p*) values from the paired *t*-test (rest vs. post-exercise) against the mean log_2_ fold changes (rest vs. post-exercise) for all the detected metabolites. Duplicate metabolites are labelled to include the metabolomics approach; POS = positive ionization mode liquid chromatography; NEG = negative ionization mode liquid chromatography; GC = gas chromatography; Tar = targeted approach

Individual acute changes in metabolite abundances in response to the standardized exercise test are illustrated with a heatmap in Fig. 2A. Significant changes were observed for metabolites involved in carbohydrate metabolism and the TCA cycle (e.g., L-lactic acid, malic acid, and glucose 6-phosphate), long- to very-long acylcarnitines (e.g., myristoylcarnitine, and L-octanoylcarnitine), amino acids/peptides (e.g., L-tryptophan and L-alanine), and purines and purine derivatives (e.g., hypoxanthine and inosine). The acute metabolic responses to exercise are also illustrated with a volcano plot (Fig. 2B), showing significant changes for metabolites involved in oxidative energy metabolism (e.g., malic acid and pyruvic acid) and acylcarnitines (e.g., trans-2-dodecenoylcarnitine and myristoleoylcarnitine). An overview of all detected metabolites and their responses to exercise is presented in Electronic Supplementary Material 5.

**Fig. 3:**
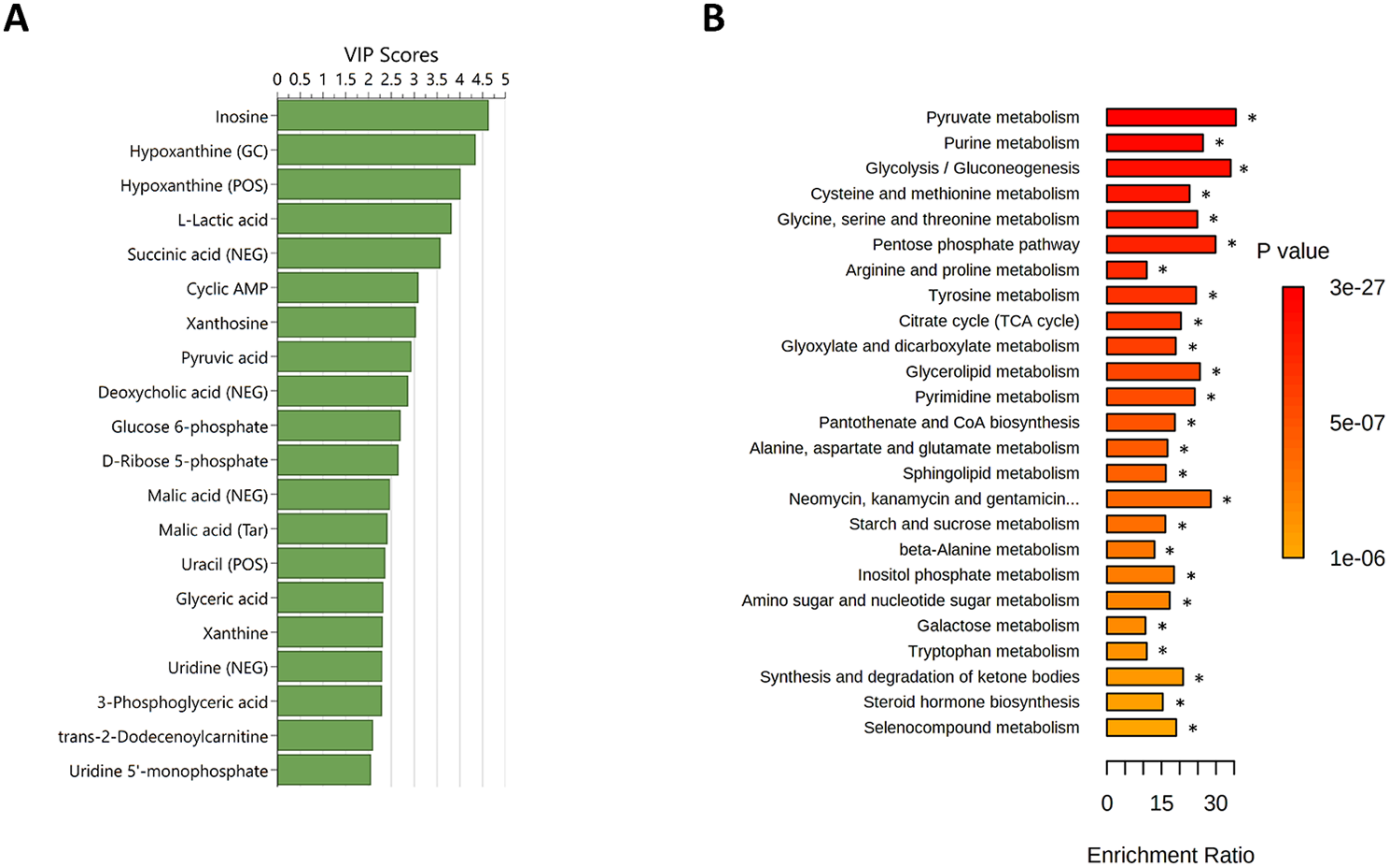
Acute changes in the metabolome in response to the standardized exercise test. **A**) Top 20 metabolites contributing to the distinction in the orthogonal partial least squares discriminant analyses based on variable influence on projection (VIP) scores. **B**) Summary plot for the quantitative enrichment analysis. Raw *p*-values are indicated by the color gradient. Duplicate metabolites are labelled to include the approach; POS = positive ionization mode liquid chromatography; NEG = negative ionization mode liquid chromatography; GC = gas chromatography; Tar = targeted approach. * significant after FDR correction

The PCA showed a clear distinction between resting and post-exercise samples over the two first principal components (see Fig. 1 in Electronic Supplementary Material 6). Similarly, OPLS-DA showed clear distinction between resting and post-exercise samples (R^2^Y = 0.978, Q^2^ = 0.962, F(4,41) = 257.276, *p* < 0.001, see Fig. 2 in Electronic Supplementary Material 6). The top 20 metabolites that contributed to the distinction in the OPLS-DA model based on VIP scores (Fig. 3A) included metabolites primarily involved in carbohydrate metabolism and the TCA cycle (e.g., succinic acid and pyruvic acid) and nucleotides and their degradation products (e.g., cAMP and inosine). The subsequent enrichment analysis showed significant differences in clusters of metabolites involved in the metabolism of several compounds, such as pyruvate, purines, and glucose (Fig. 3B).

### Differences in Resting Metabolic Phenotypes between Female and Male Athletes

**Fig. 4:**
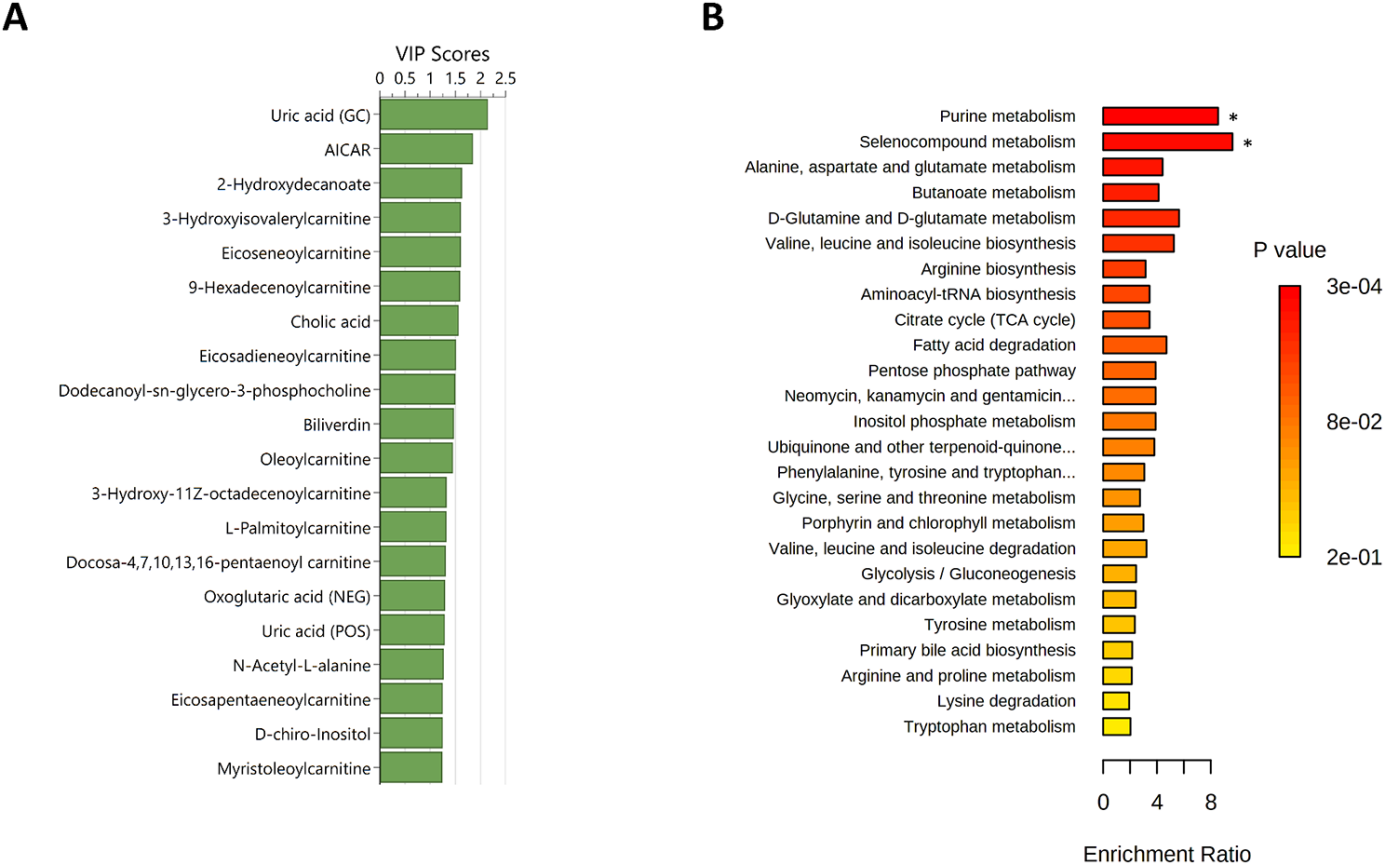
Differences in the resting metabolic phenotypes of female vs. male athletes. **A**) Top 20 metabolites contributing to the distinction in the orthogonal partial least squares discriminant analyses based on variable influence on projection (VIP) scores. **B**) Summary plot for the quantitative enrichment analysis. Raw *p*-values are indicated by the color gradient. Duplicate metabolites are labelled to include the approach; POS = positive ionization mode liquid chromatography; NEG = negative ionization mode liquid chromatography; GC = gas chromatography. * significant after FDR correction

The PCA showed no clear distinction between the resting metabolic phenotypes of the female and male athletes over the first two principal components (see Fig. 3 in Electronic Supplementary Material 6). The refined OPLS-DA model, which included 108 metabolites, showed good distinction between the sexes (R^2^Y = 0.957, Q^2^ = 0.667, F(6,16) = 5.333, *p* = 0.003, see Fig. 4 in Electronic Supplementary Material 6). The top 20 metabolites that contributed to the distinction included uric acid, AICAR (5-Aminoimidazole-4-carboxamide ribonucleotide) and 2-hydroxydecanoate (Fig. 4A). The subsequent enrichment analysis showed significant differences (after FDR correction) in purine and seleno-compound metabolism (Fig. 4B).

### Differences between Higher and Lower Peak Blood Lactate Groups

**Fig. 5:**
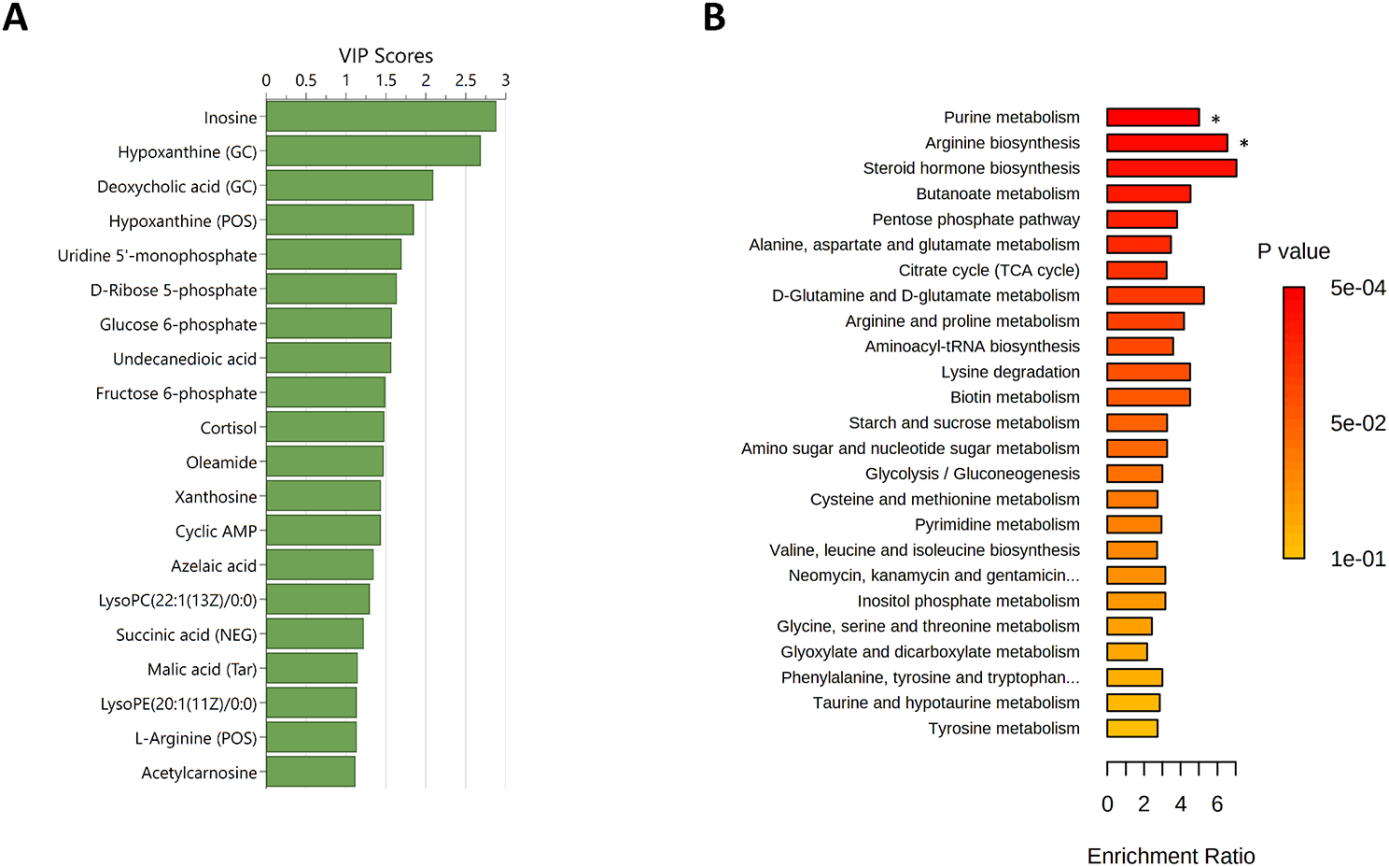
Differences in metabolic phenotype changes from before to after exercise in high vs. low peak blood lactate groups. **A**) Top 20 metabolites contributing to the distinction in the orthogonal partial least squares discriminant analyses based on variable influence on projection (VIP) scores. **B**) Summary plot for the quantitative enrichment analysis. Raw *p*-values are indicated by the color gradient. Duplicate metabolites are labelled to include the approach; POS = positive ionization mode liquid chromatography; NEG = negative ionization mode liquid chromatography; GC = gas chromatography; Tar = targeted approach. * significant after FDR correction

**Fig. 6:**
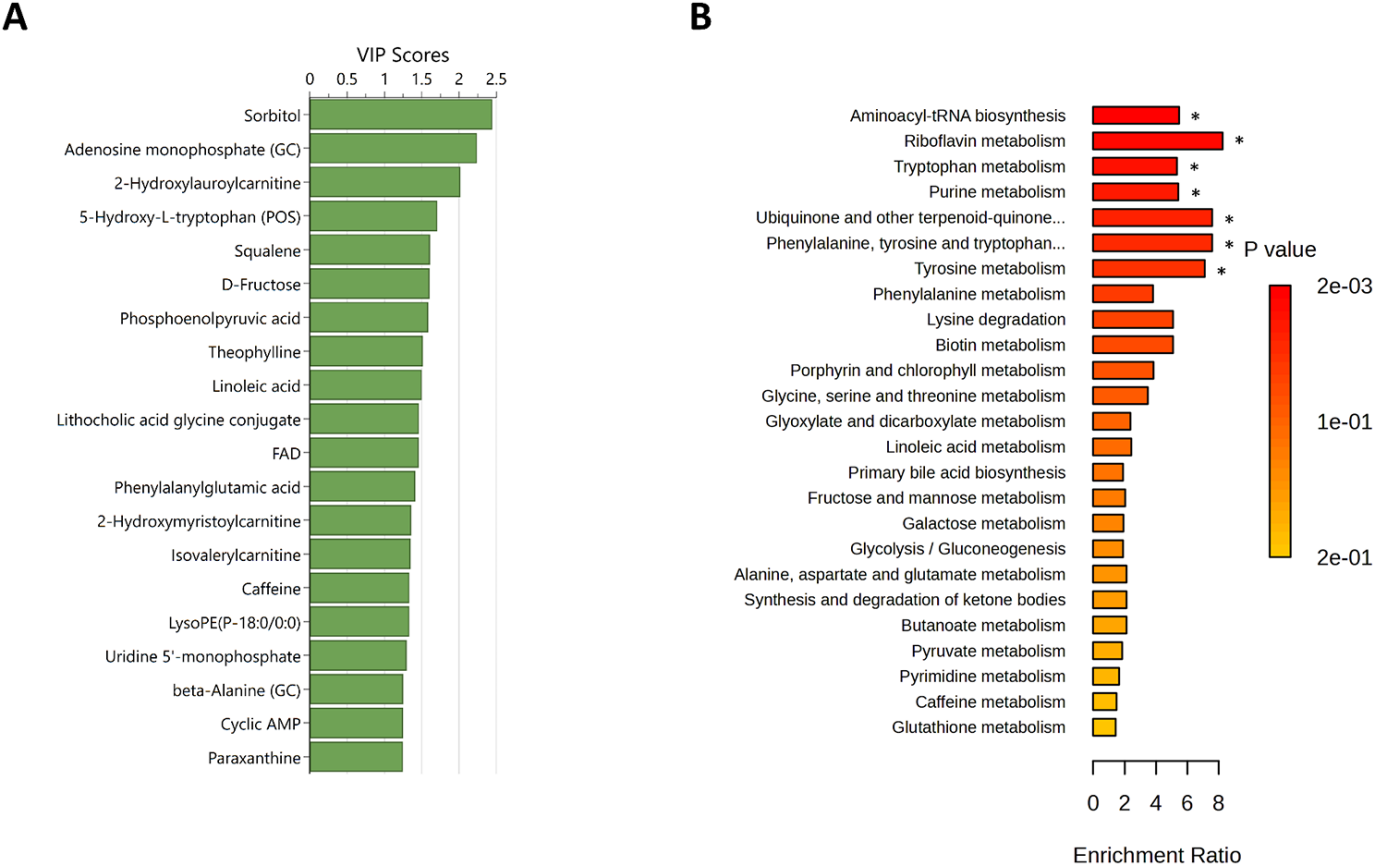
Differences in metabolic phenotype changes from before to after exercise in superior and inferior sprint skiers. **A**) Top 20 metabolites contributing to the distinction in the orthogonal partial least squares discriminant analyses based on variable influence on projection (VIP) scores. **B**) Summary plot for the quantitative enrichment analysis. Raw *p*-values are indicated by the color gradient. Duplicate metabolites are labelled to include the approach; POS = positive ionization mode liquid chromatography; GC = gas chromatography. * significant after FDR correction

The PCA showed no clear distinction between the high and low [La^−^]_peak_ groups based on changes in the metabolic phenotypes from before to after exercise over the two first principal components (se Fig. 5 in Electronic Supplementary Material 6). The refined OPLS-DA model, which included 95 metabolites, showed good distinction between the high and low [La^−^]_peak_ groups (R^2^Y = 0.900, Q^2^ = 0.663, F(4,18) = 8.868, *p* < 0.001, see Fig. 6 in Electronic Supplementary Material 6). The top 20 metabolites that contributed to the distinction included inosine, hypoxanthine, and deoxycholic acid (Fig. 5A). The subsequent enrichment analysis showed significant differences (after FDR correction) in purine metabolism and arginine biosynthesis (Fig. 5B).

### Differences between Superior and Inferior Sprint Skiers

The PCA showed no clear distinction between the superior and inferior sprint skiers based on changes in the metabolic phenotypes from before to after exercise over the two first principal components (see Fig. 5 in Electronic Supplementary Material 6). The refined OPLS-DA model included 116 metabolites and showed good distinction between superior and inferior sprint performers (R^2^Y = 0.890, Q^2^ = 0.557, F(6,16) = 3.350, *p* = 0.025, see Fig. 8 in Electronic Supplementary Material 6). The top 20 metabolites that contributed to the distinction included sorbitol, adenosine monophosphate, and 2-hydroxyleuroylcarnitine (Fig. 6A). The subsequent enrichment analysis showed significant differences (after FDR correction) in pathways related to aminoacyl-tRNA biosynthesis, riboflavin metabolism, and tryptophan metabolism. (Fig. 6B).

### Differences between Illness-Susceptible and -Non-Susceptible Athletes

**Fig. 7:**
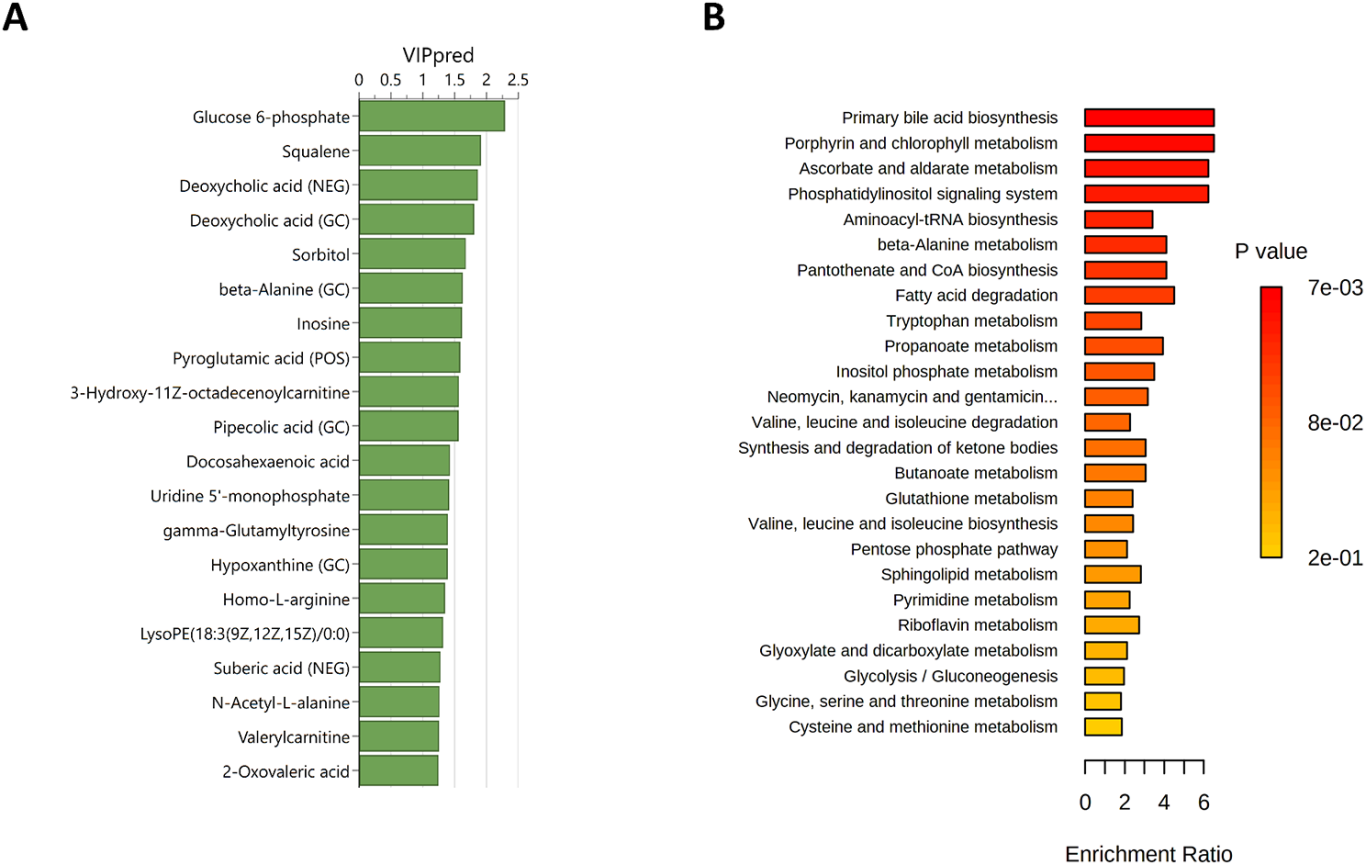
Differences in metabolic phenotype changes from before to after exercise in susceptible (i.e., ≥ 2 illness episodes in the 33-week study period) vs. non-susceptible (i.e., ≤ 1 illness episode in the 33-week study period) athletes. **A**) Top 20 metabolites contributing to the distinction in the orthogonal partial least squares discriminant analyses based on variable influence on projection (VIP) scores. **B**) Summary plot for the quantitative enrichment analysis. Raw p-values are indicated by the color gradient. Duplicate metabolites are labelled to include the approach; POS = positive ionization mode liquid chromatography; NEG = negative ionization mode liquid chromatography; GC = gas chromatography. * significant after FDR correction

The PCA showed no clear distinction between S and NS athletes based on changes in the metabolic phenotypes from before to after exercise over the two first principal components (see Fig. 9 in Electronic Supplementary Material 6). The refined OPLS-DA model included 129 metabolites and showed good distinction between S and NS athletes (R^2^Y = 0.916, Q^2^ = 0.617, F(6,15) = 4.025, *p* = 0.013, see Fig. 10 in Electronic Supplementary Material 6). The top 20 metabolites that contributed to the distinction included glucose-6-phosphate, squalene, and deoxycholic acid (Fig. 7A). The subsequent enrichment analysis showed no significant differences in the two groups after FDR correction (Fig. 7B).

## Discussion

The present study examined acute changes in the metabolic phenotypes of a group of highly-trained cross-country skiers in response to a standardized exercise test. The study also examined whether the resting metabolic phenotype, and/or acute changes in the metabolic phenotype in response to exercise, could discriminate between athlete groups with specific physiological, performance, and illness characteristics. The main findings were that: (1) a standardized exercise test led to alterations in metabolites primarily involved in carbohydrate metabolism and the TCA cycle, purine/pyrimidines, and nucleoside/nucleotides; (2) the resting metabolic phenotype was different in female and male XC skiers, with differences in uric acid, AICAR, and acylcarnitine metabolism; (3) acute changes in the metabolic phenotype could differentiate athletes with higher vs. lower [La^−^]_peak_ and superior vs. inferior sprint skiers; and (4) acute changes in the metabolic phenotype could differentiate between athletes who were susceptible vs. non-susceptible to illness.

### Acute Changes in the Metabolic Phenotype in Response to Exercise

Several studies have used metabolic phenotyping to characterize metabolic responses to acute exercise [6, 11, 25]. However, direct comparisons to previous studies are complicated due to differences in exercise protocols, sampling time points, the metabolomics platform used, and metabolite coverage [5, 9]. Nonetheless, our results align with some general trends presented in the literature. For example, metabolites (e.g., L-lactic acid and pyruvic acid) and pathways (glycolysis and TCA cycle) involved in energy metabolism were major contributors to the distinction of resting and post-exercise samples. These changes reflect the mobilization, utilization, and conversion of energy substrates (e.g., fat and carbohydrates) to meet the adenosine triphosphate (ATP) demands of the exercising muscles [9]. Moreover, during high-intensity exercise, blood glucose and muscle glycogen are the main energy sources [26], leading to increased plasma pyruvate and lactate concentrations [9, 25].

Nucleotides and their degradation products were also important in distinguishing the resting and post-exercise metabolic phenotypes. Nucleotides are essential organic molecules for energy metabolism, cell-signaling, and as substrates for deoxyribonucleic acid (DNA) and ribonucleic acid (RNA) synthesis. Phosphorylated nucleotides such as ATP and guanosine triphosphate (GTP) are usually trapped inside the cell and are rarely detected in blood or other biofluids [27]. However, in the early phase after exercise, unphosphorylated nucleotides and their degradation products, such as inosine and hypoxanthine, typically increase in blood as a consequence of the increased nucleotide breakdown during exercise [9], which is consistent with results from our study. However, while the activation of the major pathways involved in macronutrient metabolism is predictable (i.e., individuals generally exhibit similar responses that vary in magnitude), the intriguing aspect of metabolic phenotyping lies in the discovery of metabolites or pathways that can distinguish between athletes exhibiting different characteristics.

### The Resting Metabolic Phenotypes Differentiates the Sexes

In the present study, female and male athletes could be differentiated based on their resting metabolic phenotypes. This is consistent with previous studies showing differences in the fasted resting metabolic phenotypes of healthy (non-athletic) women and men for a variety of biofluids (e.g., serum, plasma, and urine) [28, 29]. Uric acid was the strongest contributor to the distinction between sexes in our OPLS-DA model, with higher concentrations in men vs. women, which is a well-known sexual dimorphism in humans [30]. Uric acid is the product of purine compound metabolism and is catalyzed by the enzyme xanthine oxidase [30]. Estradiol may contribute to this dimorphism, as estradiol has been shown to suppress xanthine oxidase activity in rat liver [31] and can decrease circulating uric acid when administered pharmacologically [32]. However, recent evidence challenges the role of estradiol as a cause of the sexual dimorphism in circulating uric acid concentration under normal physiological conditions [30].

The medium-chain fatty acid 2-hydroxydecanoate was another important contributor to the distinction of the sexes at rest in the present study, with a higher relative abundance in the female vs. male athletes. Serum concentrations of medium chain fatty acids are generally higher in women than men [28] and in our study, 10 of the top 20 metabolites contributing to the distinction of the sexes in the OPLS-DA model were acylcarnitines. Acylcarnitines are fatty acids bound to carnitine and their general role is to transport fatty acids from the cytoplasm into the mitochondria for energy production [4]. Even though acylcarnitines are produced within the cell, they can also appear in blood and other biofluids [4]. Similar to the present study, higher concentrations of acylcarnitines have been reported in healthy, non-athletic men vs. women [33]. Higher concentrations of acylcarnitines in men may be explained by a greater food intake in men compared with women [33] and differences in acylcarnitine concentrations may relate to sex-based variations in fat metabolism [34, 35], but this might not be the case in athletes [36]. Sex differences in the resting metabolic phenotypes of healthy non-athletic and athletic individuals are evident. Therefore, separate normal ranges for sexes and individual athletes are necessary to ensure correct and meaningful interpretations of the data if metabolic phenotyping for athlete monitoring is to be implemented.

Interestingly, while women and men could be distinguished at rest, it was not possible to distinguish the sexes based on the changes in metabolic phenotypes from rest to post-exercise. This observation suggests that metabolic phenotyping revealed more commonalities in the exercise response between men and women than possible sex-specific differences. One potential cause of differences in the exercise response between women and men could be menstrual cycle phase or changes in the female hormone profile, which was not controlled for in this study. However, recent evidence suggests that performance-determining variables are not influenced by menstrual cycle phase, serum estrogen, or progesterone concentrations, at least on a group level [37].

### Changes in the Metabolic Phenotype Differentiate Athletes with Higher vs. Lower peak Blood Lactate Concentrations

The high and low [La^−^]_peak_-groups could be distinguished by the changes in their metabolic phenotypes from rest to post-exercise. Inosine and hypoxanthine were the main contributors to the distinction, with higher relative abundances in the low vs. high [La^−^]_peak_-group. Correspondingly, the enrichment analysis showed a significant difference in purine metabolism between the groups. Both inosine and hypoxanthine are intermediates in the degradation of purines and purine nucleosides and their abundance is typically increased following an acute bout of exercise [5, 9]. Inosine and hypoxanthine have physiological functions that can potentially enhance endurance performance, including acting as vasodilators [38, 39]. In addition, inosine is a precursor to 2,3-diphosphoglycerate formation and could therefore contribute to a right-shift in the oxyhemoglobin dissociation curve during exercise [38]. Both mechanisms would facilitate increased oxygen delivery to the working muscles. A greater capacity for oxidative metabolism in the low [La^−^]_peak_-group, as inferred by the greater abundance of inosine in this group compared to high [La^−^]_peak_-group, support this theory.

### Changes in the Metabolic Phenotype Differentiate Superior vs. Inferior Sprint Skiers

Higher- and lower-performing sprint skiers could be distinguished by the changes in their metabolic phenotypes from rest to post-exercise. Whilst aerobic power and anaerobic capacity have been associated with superior XC sprint performance [40–42], few metabolites directly involved in the major energy pathways (e.g., glycolysis and the TCA cycle) were key contributors to the distinction in the OPLS-DA model. According to the enrichment analysis, there was a significant difference in the riboflavin metabolism pathway between the two groups. Riboflavin, or vitamin B_2_, is a water-soluble vitamin and the precursor of the coenzymes flavin mononucleotide and flavin-adenine dinucleotide, which have key functions within the TCA cycle and the electron transport chain [43]. Thus, it is plausible that riboflavin metabolism can influence endurance performance [43]. While some evidence suggests that a deficiency of certain B-complex vitamins (including riboflavin) can promote premature lactate accumulation and reduce V̇O_2peak_ [44, 45], results concerning the potential role of riboflavin in athletic performance are inconclusive [43]. Thus, further investigations are required to improve our understanding of riboflavin’s role in sports performance.

### Changes in the Metabolic Phenotype Differentiate between Illness-Susceptible vs. -Non-Susceptible Athletes

To our knowledge, this is the first study to investigate whether changes in the metabolic phenotype in response to a standardized exercise test can differentiate illness susceptibility in highly-trained endurance athletes, and the second study to investigate links between the metabolome and illnesses in athletes [13]. In the present study, athletes in the S and NS groups could be differentiated based on the changes in their metabolic phenotypes from rest to post-exercise. The five most important contributors to the distinction of S and NS were glucose-6-phosphate, squalene, deoxycholic acid, sorbitol, and beta-alanine. However, the enrichment analysis was unable to detect any significant differences in metabolic pathway activity between groups. Hence, further investigation is warranted to determine whether changes in athletes’ metabolic phenotypes after exercise can be used to identify illness susceptibility in highly-trained athletes.

The S and NS athletes could not be distinguished based on resting metabolic phenotypes. This contrasts with the findings of Cuthbertson et al. [13], who reported that the resting plasma metabolomic phenotypes differed between highly-susceptible compared to non-susceptible athletes (i.e., ≥ 4 vs. ≤ 3 RTIs over the 18-month study period, respectively). An explanation for the inconsistent results might be the relatively small sample size of the present study (*n* = 23), which may have led to true positive results being missed due to insufficient statistical power. Another explanation might be the low threshold for inclusion in the S group (2 illnesses in 18 months) in the present study, or that different sampling materials were used (i.e., whole blood in the present study vs. venous plasma by Cuthbertson et al. [13]). The ability to identify athletes susceptible to illness from resting samples alone would be beneficial from a practical standpoint, as resting samples would be easy to implement without interrupting the athletes’ training and recovery routines. On the other hand, given the complex interplay between metabolic and immune responses, and the strong acute reactivity of immune responses to exercise stress [46, 47], we could speculate that alterations in immunometabolic responses to exercise have the potential to precede changes that manifest at rest. Therefore, further research is warranted to elucidate whether the resting metabolic phenotype and immunometabolic responses to exercise can be used to monitor illness susceptibility in elite endurance athletes.

### Limitations

The present study was designed around a standardized exercise test that is typically completed biannually by the participating athletes as part of their routine monitoring. Hence, a major strength is that the data reflects the athletes’ preparations for and responses to a test used in an applied setting. The relatively small sample of 23 athletes may have resulted in true positive results being missed due to insufficient statistical power [25]. This is a recurring issue in research with high-performing athletes [48]. However, the sample size in the present study was similar to that of previous studies applying the same statistical approach with athlete samples [19, 49]. A small sample size can also increase the risk of overfitting [50]. To mitigate this risk, we cross-validated our models using CV-ANOVA as recommended by Worley and Powers [51] to decrease the risk of OPLS-DA models forcing distinction between groups by modelling noise. Moreover, due to the relatively small sample size, data from both sexes were combined in the analyses to increase statistical power. Sex differences in the metabolic phenotypes of humans are well documented in the scientific literature [28–30, 52, 53]. Despite the observed differences at baseline, the current analyses suggest that there are more commonalities than differences between sexes when it comes to the exercise response. Furthermore, the dietary intake of the athletes prior to exercise was not standardized in the present study. Hence, individual differences in dietary composition and timing, and the use of supplements, medications, and/or other ergogenic aids may have influenced the observed metabolic phenotypes [9, 25, 54]. While standardizing the dietary intake of the athletes prior to sampling might have reduced the noise in our results, the ecological validity of the study would have been compromised.

The application of MS-based metabolomics for analysis of capillary whole blood samples is relatively novel and warrants evaluation. MS-based metabolite phenotyping has been increasing in both popularity and its range of applications in recent years, but methodological challenges remain in feature identification in untargeted analyses that can result in a lack of reproducibility between studies [55, 56]. Therefore, it is important that our results are interpreted with caution and in the context of other similar results in the literature, and that key findings are validated in future studies. In the present study, metabolic phenotypes were also determined from low-volume capillary whole blood samples. Whole blood samples were used as they are practical and minimally invasive. That is, due to routine testing of blood lactate concentrations during the athletes’ physiological tests, no additional sampling was required to collect samples for metabolic phenotyping. On the one hand, whole blood samples represent an additional cellular component of blood and could result in a more comprehensive signal of the metabolic phenotype; on the other hand capillary blood is less well validated than plasma/serum and potentially more complex to interpret, with the potential for some continued metabolic activity from the cellular component after sampling [57]. For this reason, samples were frozen as soon as possible after collection. Recent studies have shown good coverage of the metabolite response to exercise in whole blood capillary samples and dried blood spots analyzed with MS-based methods [58, 59]. Additionally, only modest correlations between levels of muscle and plasma/serum metabolites have been reported [60]. Thus, the whole blood metabolic phenotype may not accurately reflect changes in the muscle metabolome [5]. However, muscle metabolic phenotyping requires muscle biopsies, therefore limiting its application within a practical setting in athletes.

## Conclusions

Changes in the metabolomic phenotype from rest to post-exercise in highly-trained XC skiers included changes in metabolites and pathways primarily involved in energy, purine, and nucleotide metabolism. The resting metabolomic phenotype could differentiate between female and male athletes, while acute changes in the metabolic phenotype from rest to post-exercise could differentiate between athletes with higher vs. lower [La^−^]_peak_ and superior vs. inferior sprint skiers. Acute changes in the metabolic phenotype could also distinguish athletes grouped according to illness susceptibility. Taken together, this pilot study suggests that metabolic phenotyping may have potential to help identify new pathways or biomarkers associated with exercise capacity or illness susceptibility; information that could be particularly valuable to optimize performance and protect athletes’ health.

In the present study several findings diverged from studies that have examined the metabolic phenotype among endurance athletes and their associations with performance and health. Thus, further studies including targeted metabolomics methods are necessary to confirm the present findings. In addition, longitudinal studies applying metabolic phenotyping at regular intervals are needed to establish normal ranges, and to determine natural variations in metabolite changes throughout the days and across a season, before implementing the method as part of a regular athlete monitoring program.

## Electronic Supplementary Material

Below is the link to the electronic supplementary material.

Supplementary Material 1

Supplementary Material 2

Supplementary Material 3

Supplementary Material 4

Supplementary Material 5

Supplementary Material 6

## Data Availability

The datasets generated and/or analyzed during the current study are not publicly available because the data contain information that could compromise research participants’ privacy and/or consent. Data are available from the corresponding author upon reasonable request.

## Abbreviations

AICAR: 5-Aminoimidazole-4-carboxamide ribonucleotide
ATP: Adenosine triphosphate
BCAA: Branched-chain amino acid
CV-ANOVA: Cross-validation analysis of variance
DNA: Deoxyribonucleic acid
FDR: False discovery rate
FIS: International Ski Federation
GC-MS: Gas chromatography-mass spectrometry
GTP: Guanosine triphosphate
[La^−^]: Blood lactate concentration
[La^−^]_max_: Maximal blood lactate concentration
[La^−^]_peak_: Peak blood lactate concentration
LC-MS: Liquid chromatography-mass spectrometry
NS: Non-susceptible (to illness)
OPLS-DA: Orthogonal partial least squares discriminant analyses
OSTRC-H2: Oslo Sports Trauma Research Center Questionnaire on Health Problems
PCA: Principal component analyses
Q^2^: Cumulative fraction of the variation of the response predicted by the model up to the specified component
RNA: Ribonucleic acid
RTIs: Respiratory tract infections
R^2^Y: Cumulative fraction of the variation of the response explained by the model up to the specified component
S: Susceptible (to illness)
SD: Standard deviation
TCA: Tricarboxylic acid
TT: Time trial
VIP: Variable influence on projection
V̇O_2peak_: Peak oxygen consumption
%V̇O_2peak_: Percentage of peak oxygen consumption
XC: Cross-country
