# Supplementary material for: Metabolic Phenotyping from Whole-Blood Responses to a Standardized Exercise Test May Discriminate for Physiological, Performance, and Illness Outcomes: A Pilot Study in Highly-Trained Cross-Country Skiers
Source: Sports Med Open. 2024 Sep 18;10:99. doi: 10.1186/s40798-024-00770-0 (PMC11408465; doi:10.1186/s40798-024-00770-0)
Supplement: Supplementary file 1 — Supplementary Material 1 [file 40798_2024_770_MOESM1_ESM.pdf]

Electronic Supplementary Material 1: Metabolic Phenotyping Procedure

Journal: Sports Medicine Open

Title: Metabolic phenotyping from whole-blood responses to a standardized exercise test may discriminate for physiological, performance, and illness outcomes: A pilot study in highly-trained cross-country skiers

Authors: Øyvind Karlsson[1], Andrew D. Govus[2], Kerry McGawley[1] & Helen G. Hanstock[1]

Affiliations:

1: Swedish Winter Sports Research Centre, Department of Health Sciences, Mid Sweden University, Östersund, Sweden

2: Department of Sport, Exercise, and Nutrition, La Trobe University, Melbourne, Victoria, Australia

Sample Preparation

Sample preparation of the capillary blood samples was performed according to A et al.[1] In detail, 360 µL of extraction buffer (90:10, v/v, methanol/water) including internal standards for both gas chromatography-mass spectrometry (GC-MS) and liquid chromatography-mass spectrometry (LC-MS) were added to 40 µL of capillary blood. The sample was shaken at 30 Hz for 2 minutes in a mixer mill and proteins were precipitated at +4°C on ice. The sample was centrifuged at 14 000 rpm and +4°C for 10 min. The supernatant, 150 µL for LC-MS analysis and 25 µL to GC-MS analysis, was transferred to microvials and evaporated to dryness in a speed-vac concentrator. The samples were stored at −80°C until they were analyzed.

A small aliquot of the remaining supernatants was pooled and used to create quality control (QC) samples. LC tandem mass spectrometry (MSMS) analysis was run on the QC samples for metabolite identification purposes. The samples were analyzed in batches according to a randomized run order on both GC-MS and LC-MS.

GC-MS Analysis

For GC-MS analysis a 9-point calibration curve (aconitic acid, a-keto-glutaric acid, alanine, aspargine, aspartic acid, citric acid, fumaric acid, gamma-amino-butyric acid, glutamine, glutamic acid, glucose, glycine, homoserine, isocitric acid, isoleucine, lactic acid, lysine, malic acid, ornithine, proline, serine, shikimic acid, succinic acid, sucrose, threonine, tryptophan, urea, valine) spanning from 25–6400 $pg \cdot \mu L^{-1}$ was prepared by serial dilutions and spiked with internal standards.

Derivatization and GC-MS analysis were performed as described previously.[1,2] The final derivatized volume was 45 µL. The 9-point calibration curve was run in parallel with the

samples. 0.5 µL of the derivatized sample was injected in splitless mode by a L-PAL3 autosampler (CTC Analytics AG, Switzerland) into an Agilent 7890B gas chromatograph equipped with a 10 m x 0.18 mm fused silica capillary column with a chemically bonded 0.18 µm Rxi-5 Sil MS stationary phase (Restek Corporation, Bellfonte, Pennsylvania, USA). The injector temperature was 270 °C, the purge flow rate was 20 $mL{\cdot}min^{-1}$ and the purge was turned on after 60 s. The gas flow rate through the column was 1 $mL{\cdot}min^{-1}$, the column temperature was held at 70 °C for 2 min, then increased by 40 $°C{\cdot}min^{-1}$ to 320 °C, and held there for 2 min. The column effluent was introduced into the ion source of a Pegasus BT time-of-flight mass spectrometer, GC/TOFMS (Leco Corp., St Joseph, Michigan, USA). The transfer line and the ion source temperatures were 250 °C and 200 °C, respectively. Ions were generated by a 70 eV electron beam at an ionization current of 2.0 mA, and 30 $spectra{\cdot}s^{-1}$ were recorded in the mass range m/z 50–800. The acceleration voltage was turned on after a solvent delay of 150 s. The detector voltage was 1800–2300 V.

LC-MS Analysis

Before LC-MS analysis the sample was re-suspended in 10 + 10 µL methanol and water. Each batch of samples was firstly analyzed in positive mode. After all samples within a batch had been analyzed, the instrument was switched to negative mode and a second injection of each sample was analyzed. The chromatographic separation was performed on an Agilent 1290 Infinity UHPLC-system (Agilent Technologies, Waldbronn, Germany). 2 µL of each sample were injected onto an Acquity UPLC HSS T3, 2.1 x 50 mm, 1.8 µm C18 column in combination with a 2.1 mm x 5 mm, 1.8 µm VanGuard precolumn (Waters Corporation, Milford, MA, USA) held at 40 °C. The gradient elution buffers were A (H_2O, 0.1% formic acid) and B (75:25, v/v, acetonitrile/2-propanol, 0.1% formic acid), and the flow rate was 0.5 $mL{\cdot}min^{-1}$. The compounds were eluted with a linear gradient consisting of 0.1–10% B over 2 min, B was increased to 99%

over 5 min and held at 99% for 2 minutes; B was decreased to 0.1% for 0.3 min and the flow rate was increased to 0.8 mL·min^{-1} for 0.5 min; these conditions were held for 0.9 min, after which the flow rate was reduced to 0.5 mL·min^{-1} for 0.1 min before the next injection.

The compounds were detected with an Agilent 6546 Q-TOF mass spectrometer (Agilent Technologies, Waldbronn, Germany) equipped with a jet stream electrospray ion source operating in positive or negative ion mode. The settings were kept identical between the modes, with the exception of the capillary voltage. A reference interface was connected for accurate mass measurements; the reference ions purine (4 μM) and HP-0921 (Hexakis(1H, 1H, 3H-tetrafluoropropoxy)phosphazine) (1 μM) were infused directly into the MS at a flow rate of 0.05 mL·min^{-1} for internal calibration, and the monitored ions were purine m/z 121.05 and m/z 119.03632; HP-0921 m/z 922.0098 and m/z 966.000725 for positive and negative mode, respectively. The gas temperature was set to 150 °C, the drying gas flow to 8 L·min^{-1} and the nebulizer pressure 35 psig. The sheath gas temperature was set to 350 °C and the sheath gas flow 11 L·min^{-1}. The capillary voltage was set to 4000 V in positive ion mode, and to 4000 V in negative ion mode. The nozzle voltage was 300 V. The fragmentor voltage was 120 V, the skimmer 65 V and the OCT 1 RF Vpp 750 V. The collision energy was set to 0 V. The m/z range was 70–1700, and data was collected in centroid mode with an acquisition rate of 4 scans·s^{-1} (1977 transients/spectrum).

Data Processing

For the GC-MS data, all non-processed MS files from the metabolic analysis were exported from the ChromaTOF software in NetCDF format to MATLAB® R2020a (Mathworks, Natick, Masachusetts, USA), where all data pre-treatment procedures, such as baseline correction, chromatogram alignment, data compression and Multivariate Curve Resolution were

performed. The extracted mass spectra were identified by comparisons of their retention index and mass spectra with libraries of retention time indices and mass spectra.[3] Mass spectra and retention index comparison was performed using NIST MS 2.2 software (National Institute of Standards and Technology, Gaithersburg, Maryland, USA). Annotation of mass spectra was based on reverse and forward searches in the library. Masses and ratio between masses indicative of a derivatized metabolite were especially notified. The mass spectrum with the highest probability indicative of a metabolite and the retention index between the sample and library for the suggested metabolite was ± 5 (usually less than 3). The deconvoluted "peak" was annotated as an identification of a metabolite. Calculations of the absolute quantification were performed for the compounds included in the calibration curve using Excel (Microsoft, Redmond, Washington, USA) software.

For the LC-MS data, all data processing was performed using the Agilent Masshunter Profinder version B.10.00 (Agilent Technologies Inc., Santa Clara, California, USA). The data pre-processing was performed in a target fashion. A pre-defined list (Table 1) of metabolites commonly found in plasma and serum were searched for using the Batch Targeted feature extraction in Masshunter Profinder. An in-house LC-MS library built up by authentic standards run on the same system with the same chromatographic and MS settings, were used for the targeted processing. The identification of the metabolites was based on MS, MSMS and retention time information. Batch Recursive Feature Extraction algorithm within Masshunter Profinder was used for the untargeted data pre-processing.

Table 1 Metabolites searched for in the targeted feature extraction. Metabolites are listed in no particular order.

Metabolite	Class/Pathway
L-Lactic acid	Glycolysis
L-Alanine	Amino acid
L-Valine	Amino acid
Urea	Amino acid breakdown
L-Isoleucine	Amino acid
L-Proline	Amino acid
Glycine	Amino acid
Succinic acid	TCA cycle
Fumaric acid	TCA cycle
L-Serine	Amino acid
L-Threonine	Amino acid
Malic acid	TCA cycle
L-Aspartic acid	Amino acid
gamma-Aminobutyric acid	Inhibitory neurotransmitter/Glutamate metabolism
Oxoglutaric acid	TCA cycle
D-Glutamic acid	Amino acid
cis-Aconitic acid*	TCA cycle
Shikimic acid*	Organic compounds
Citric acid	TCA cycle
Ornithine	Amino acid
Isocitric acid	TCA cycle
D-Glucose	Glycolysis
L-Lysine	Amino acid
L-Tryptophan	Amino acid
Glucose 6-phosphate	Glycolysis
Sucrose	Glycogenosis/Galactose Metabolism

Notes: *: Not quantified
Abbreviations: TCA = tricarboxylic acid

Chemicals

Solvents: Methanol, HPLC-grade was obtained from Fischer Scientific (Waltham, Massachusetts, USA); Chloroform, Suprasolv for GC was obtained from Merck (Darmstadt, Germany); Acetonitrile, HPLC-grade was obtained from Fischer Scientific (Waltham, Massachusetts, USA); 2-Propanol, HPLC-grade was obtained from VWR (Radnor, PA, USA) H_2O, Milli-Q Direct Laboratory Water Purification system (Merck KGaA, Darmstadt, Germany). Reference and tuning standards: Purine, 4 µM, Agilent Technologies (Santa Clara, California, USA); HP-0921 (Hexakis(1H, 1H, 3H-tetrafluoropropoxy)phosphazine), 1 µM, Agilent Technologies (Santa Clara, California, USA); Calibrant, ESI-TOF, ESI-L Low Concentration Tuning Mix, Agilent Technologies (Santa Clara, California, USA); HP-0321 (Hexamethoxyphosphazine), 0.1 mM, Agilent Technologies (Santa Clara, California, USA). Stable isotopes internal standards: LC-MS internal standards: 13C9-Phenylalanine, 13C3-Caffeine, D4-Cholic acid, D8-Arachidonic Acid, 13C9-Caffeic Acid were obtained from Sigma (St. Louis, MO, USA). GC-MS internal standards: L-proline-13C5, alpha-ketoglutarate-13C4, myristic acid-13C3, cholesterol-D7, Fumaric acid (13C4), L-Malic acid (13C4), GABA (13C4), tryptophan (D8), Glycine (13C2-15N) were obtained from Cambridge Isotope Laboratories (Andover, Massachusetts, USA). Succinic acid-D4, salicylic acid-D6, L-glutamic acid-13C5,15N, putrescine-D4, hexadecanoic acid-13C4, D-glucose-13C6, D-sucrose-13C12 were obtained from Sigma (St. Louis, Missouri, USA). The calibration curve standards (aconitic acid, a-keto-glutaric acid, alanine, aspargine, aspartic acid, citric acid, fumaric acid, gamma-amino-butyric acid, glutamine, glutamic acid, glucose, glycine, homoserine, isocitric acid, isoleucine, lactic acid, lysine, malic acid, ornithine, proline, serine, shikimic acid, succinic acid, sucrose, threonine, tryptophan, urea, valine) were all purchased from Sigma (St. Louis, Missouri, USA).

References

1. Gullberg J, Jonsson P, Nordström A, et al. Design of experiments: an efficient strategy to identify factors influencing extraction and derivatization of Arabidopsis thaliana samples in metabolomic studies with gas chromatography/mass spectrometry. *Anal Biochem* 2004;331:283-95. doi: 10.1016/j.ab.2004.04.037
2. A J, Trygg J, Gullberg J, Johansson AI, Jonsson P, Antti H, Marklund SL & Moritz T. Extraction and GC/MS analysis of the human blood plasma metabolome. Anal Chem 2005;77:8086-8094.
3. Schauer N, Steinhauser D, Strelkov S, et al. GC-MS libraries for the rapid identification of metabolites in complex biological samples. FEBS Lett 2005;579:1332-1337
