# Supplementary material for: Metabolic Phenotyping from Whole-Blood Responses to a Standardized Exercise Test May Discriminate for Physiological, Performance, and Illness Outcomes: A Pilot Study in Highly-Trained Cross-Country Skiers
Source: Sports Med Open. 2024 Sep 18;10:99. doi: 10.1186/s40798-024-00770-0 (PMC11408465; doi:10.1186/s40798-024-00770-0)
Supplement: Supplementary file 2 — Supplementary Material 2 [file 40798_2024_770_MOESM2_ESM.pdf]

Electronic Supplementary Material 2: Non-Significant Models

Journal: Sports Medicine Open

Title: Metabolic phenotyping from whole-blood responses to a standardized exercise test may discriminate for physiological, performance, and illness outcomes: A pilot study in highly-trained cross-country skiers

Authors: Øyvind Karlsson[1], Andrew D. Govus[2], Kerry McGawley[1] & Helen G. Hanstock[1]

Affiliations:

1: Swedish Winter Sports Research Centre, Department of Health Sciences, Mid Sweden University, Östersund, Sweden

2: Department of Sport, Exercise, and Nutrition, La Trobe University, Melbourne, Victoria, Australia

Overview of non-significant orthogonal partial least squares discriminant analysis models according to differentiator. Resting = models run on resting samples; Post-exercise = models run on $\log_2$ fold-change data (resting to post-exercise).

**Model**	**Components**	**n**	**R^2**	**Q^2**	**p**
Resting					
TT performance	1+0+0	22	0.719	-0.123	> 0.999
$[La^-]_{peak}$	*				
$\dot{V}O_{2abs}$	1+7+0	23	0.999	0.268	0.973
$\dot{V}O_{2rel}$	1+0+0	23	0.619	0.110	0.310
Speed@2mmol	*				
Speed@4mmol	*				
$\%\dot{V}O_{2peak}$@2mmol	1+0+0	23	0.538	-0.161	> 0.999
$\%\dot{V}O_{2peak}$@4mmol	*				
Distance performance	*				
Sprint performance	1+0+0	23	0.508	-0.073	> 0.999
Illness	1+0+0	22	0.487	-0.117	> 0.999
Post-exercise					
Sex	*				
TT performance	*				
$[La^-]_{peak}$	1+7+0	23	1	0.582	0.414
$\dot{V}O_{2abs}$	1+0+0	23	0.633	0.039	0.670
$\dot{V}O_{2rel}$	*				
Speed@2mmol	1+0+0	23	0.543	0.047	0.621
Speed@4mmol	1+0+0	22	0.509	0.075	0.456
$\%\dot{V}O_{2peak}$@2mmol	1+0+0	23	0.543	0.047	0.621
$\%\dot{V}O_{2peak}$@4mmol	1+0+0	22	0.528	0.072	0.491
Distance performance	1+0+0	23	0.522	0.012	0.886
Sprint performance	1+2+0	23	0.929	0.304	0.357
Illness susceptibility[#]	1+2+0	22	0.953	0.341	0.318

Notes: * model did not converge: ; [#]: Susceptible athletes were defined as those reporting ≥ 2 illness episodes throughout the 33-week study period, while non-susceptible athletes were defined as reporting ≤ 1 illness episode.
Abbreviations: TT = time trial; $[La^-]_{peak}$ = peak exercise blood lactate concentration; $\dot{V}O_{2abs}$ = absolute peak oxygen consumption; $\dot{V}O_{2rel}$ = relative peak oxygen consumption; Speed@2mmol = speed at a blood lactate concentration of 2 $mmol \cdot L^{-1}$; Speed@4mmol = speed at a blood lactate concentration of 4 $mmol \cdot L^{-1}$; $\%\dot{V}O_{2peak}$@2mmol = percent of $\dot{V}O_{2peak}$ at a blood lactate concentration of 2 $mmol \cdot L^{-1}$; $\%\dot{V}O_{2peak}$@4mmol = percent of $\dot{V}O_{2peak}$ at a blood lactate concentration of 4 $mmol \cdot L^{-1}$
