# Supplementary material for: Metabolic Phenotyping from Whole-Blood Responses to a Standardized Exercise Test May Discriminate for Physiological, Performance, and Illness Outcomes: A Pilot Study in Highly-Trained Cross-Country Skiers
Source: Sports Med Open. 2024 Sep 18;10:99. doi: 10.1186/s40798-024-00770-0 (PMC11408465; doi:10.1186/s40798-024-00770-0)
Supplement: Supplementary file 3 — Supplementary Material 3 [file 40798_2024_770_MOESM3_ESM.pdf]

Electronic Supplementary Material 3: Non-Significant Refined Models

Journal: Sports Medicine Open

Title: Metabolic phenotyping from whole-blood responses to a standardized exercise test may discriminate for physiological, performance, and illness outcomes: A pilot study in highly-trained cross-country skiers

Authors: Øyvind Karlsson[1], Andrew D. Govus[2], Kerry McGawley[1] & Helen G. Hanstock[1]

Affiliations:

1: Swedish Winter Sports Research Centre, Department of Health Sciences, Mid Sweden University, Östersund, Sweden

2: Department of Sport, Exercise, and Nutrition, La Trobe University, Melbourne, Victoria, Australia

Overview of non-significant refined orthogonal partial least squares discriminant analysis models (i.e., models where metabolites with a variable inflation factor < 1 from the initial model were removed) according to differentiator. Resting = models run on resting samples; Post-exercise = models run on log_2 fold-change data (resting to post-exercise).

**Model**	**Components**	**n**	**R^2**	**Q^2**	**p**
<u>Resting</u>					
TT performance	1+0+0	22	0.535	0.207	0.111
$[La^-]_{peak}$	*				
$\dot{V}O_{2abs}$	1+1+0	23	0.793	0.124	0.642
$\dot{V}O_{2rel}$	1+0+0	23	0.365	0.182	0.134
Speed@2mmol	*				
Speed@4mmol	*				
%$\dot{V}O_{2peak}$@2mmol	1+0+0	23	0.290	0.088	0.397
%$\dot{V}O_{2peak}$@4mmol	*				
Distance performance	*				
Sprint performance	1+0+0	23	0.285	0.101	0.344
Illness	1+0+0	22	0.275	0.060	0.554
<u>Post-exercise</u>					
Sex	*				
TT performance	*				
$\dot{V}O_{2abs}$	1+0+0	23	0.419	0.140	0.224
$\dot{V}O_{2rel}$	*				
Speed@2mmol	1+0+0	23	0.318	0.108	0.317
Speed@4mmol	1+0+0	22	0.260	0.044	0.652
%VO_{2peak}@2mmol	1+0+0	23	0.318	0.108	0.317
%VO_{2peak}@4mmol	1+0+0	22	0.268	0.063	0.541
Distance performance	1+0+0	23	0.324	0.151	0.194

Notes: *: model did not converge.

Abbreviations: TT = time trial; $[La^-]_{peak}$ = post exercise blood lactate concentration; $\dot{V}O_{2abs}$ = absolute peak oxygen consumption; $\dot{V}O_{2rel}$ = relative peak oxygen consumption; Speed@2mmol = speed at a blood lactate concentration of 2 $mmol \cdot L^{-1}$; Speed@4mmol = speed at a blood lactate concentration of 4 $mmol \cdot L^{-1}$; %$\dot{V}O_{2peak}$@2mmol = percent of $\dot{V}O_{2peak}$ at a blood lactate concentration of 2 $mmol \cdot L^{-1}$; %$\dot{V}O_{2peak}$@4mmol = percent of $\dot{V}O_{2peak}$ at a blood lactate concentration of 4 $mmol \cdot L^{-1}$
