# Supplementary material for: Metabolic Phenotyping from Whole-Blood Responses to a Standardized Exercise Test May Discriminate for Physiological, Performance, and Illness Outcomes: A Pilot Study in Highly-Trained Cross-Country Skiers
Source: Sports Med Open. 2024 Sep 18;10:99. doi: 10.1186/s40798-024-00770-0 (PMC11408465; doi:10.1186/s40798-024-00770-0)
Supplement: Supplementary file 6 — Supplementary Material 6 [file 40798_2024_770_MOESM6_ESM.pdf]

Electronic Supplementary Material 6: Score Plots and Model Diagnostics

Journal: Sports Medicine Open

Title: Metabolic phenotyping from whole-blood responses to a standardized exercise test may discriminate for physiological, performance, and illness outcomes: A pilot study in highly-trained cross-country skiers

Authors: Øyvind Karlsson[1], Andrew D. Govus[2], Kerry McGawley[1] & Helen G. Hanstock[1]

Affiliations:

1: Swedish Winter Sports Research Centre, Department of Health Sciences, Mid Sweden University, Östersund, Sweden

2: Department of Sport, Exercise, and Nutrition, La Trobe University, Melbourne, Victoria, Australia

Principal Component Score Plots and Orthogonal Partial Least Squares Discriminant Analysis Model Diagnostics.

Figure 1

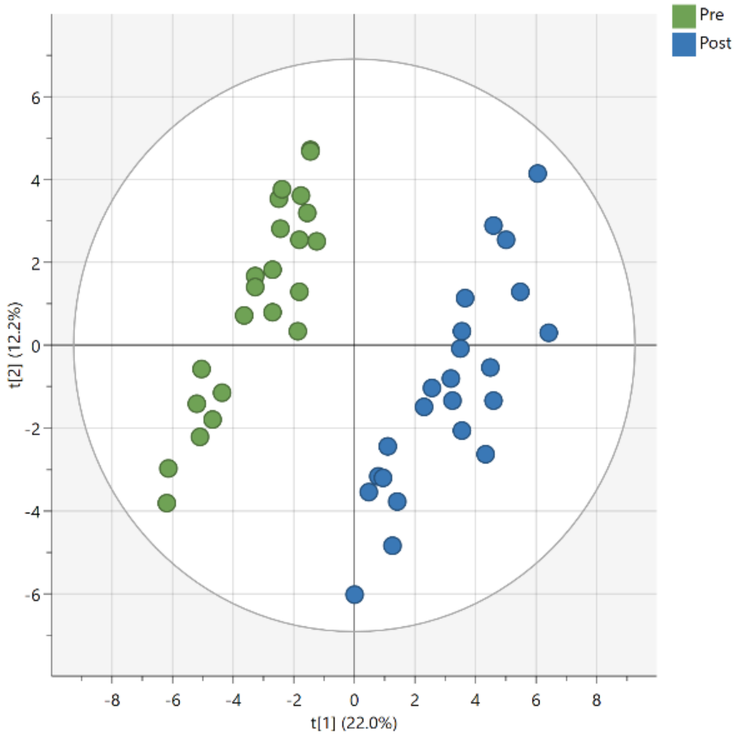

Figure 1 Principal component analysis showing rest (pre) vs. post-exercise samples.

Figure 2

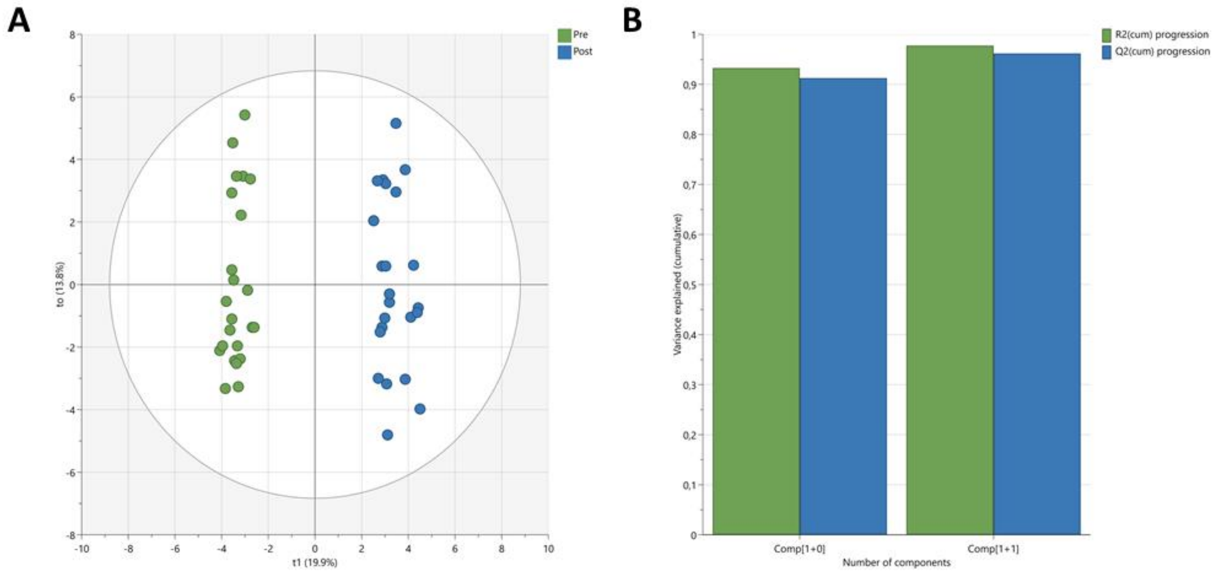

Figure 2 Model fit of the orthogonal partial least squares discriminant analysis (OPLS-DA) separating resting (pre) and post-exercise samples. A) Scores plot. B) Cumulative percent of the variation of the response explained by the model after the last component in the OPLS-DA.

Figure 3

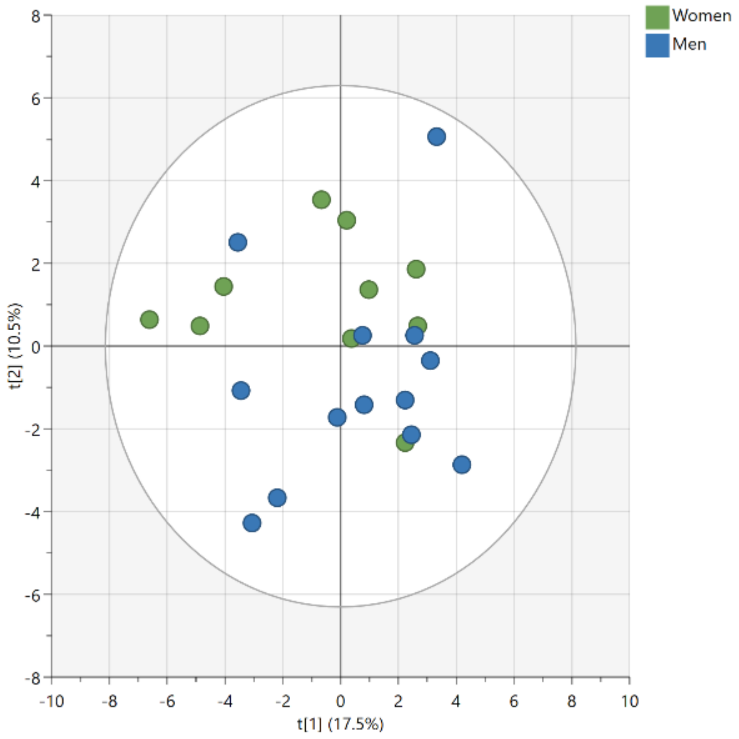

Figure 3 Principal component analysis showing the resting metabolic profiles of women vs. men.

Figure 4

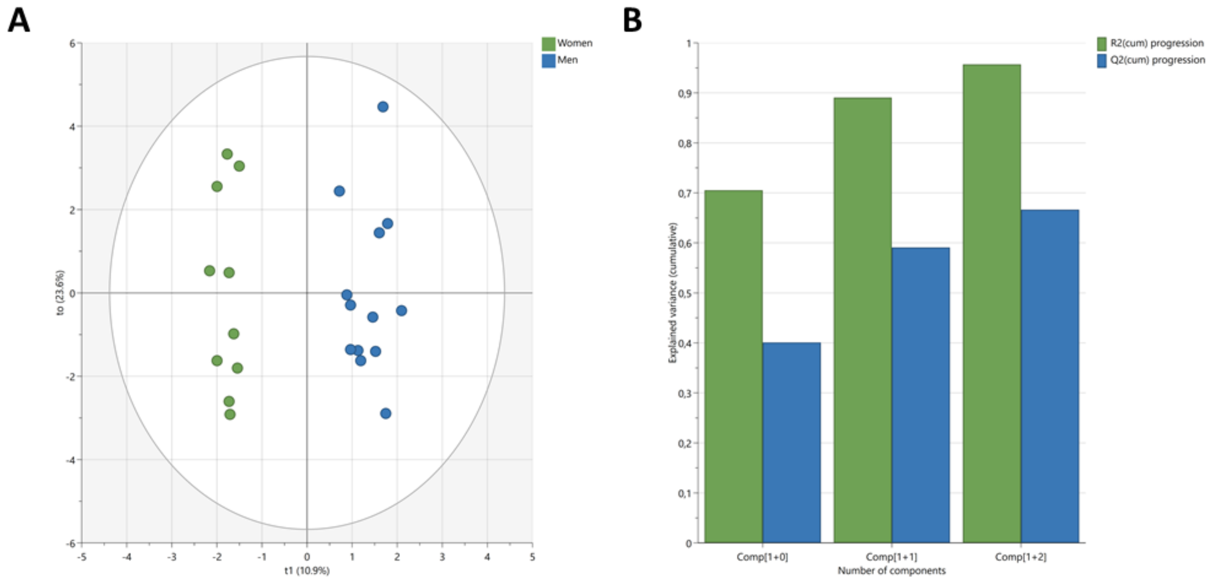

Figure 4 Model fit of the orthogonal partial least squares discriminant analysis (OPLS-DA) separating the resting metabolic profiles of women and men. A) Scores plot. B) Cumulative percent of the variation of the response explained by the model after the last component in the OPLS-DA.

Figure 5

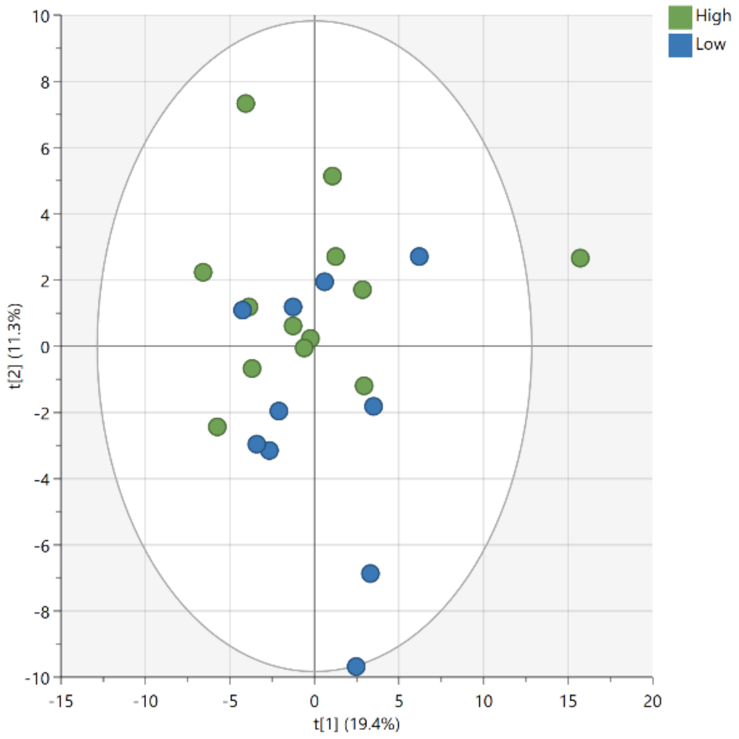

Figure 5 Principal component analysis showing high and low post-exercise blood lactate concentration groups.

Figure 6

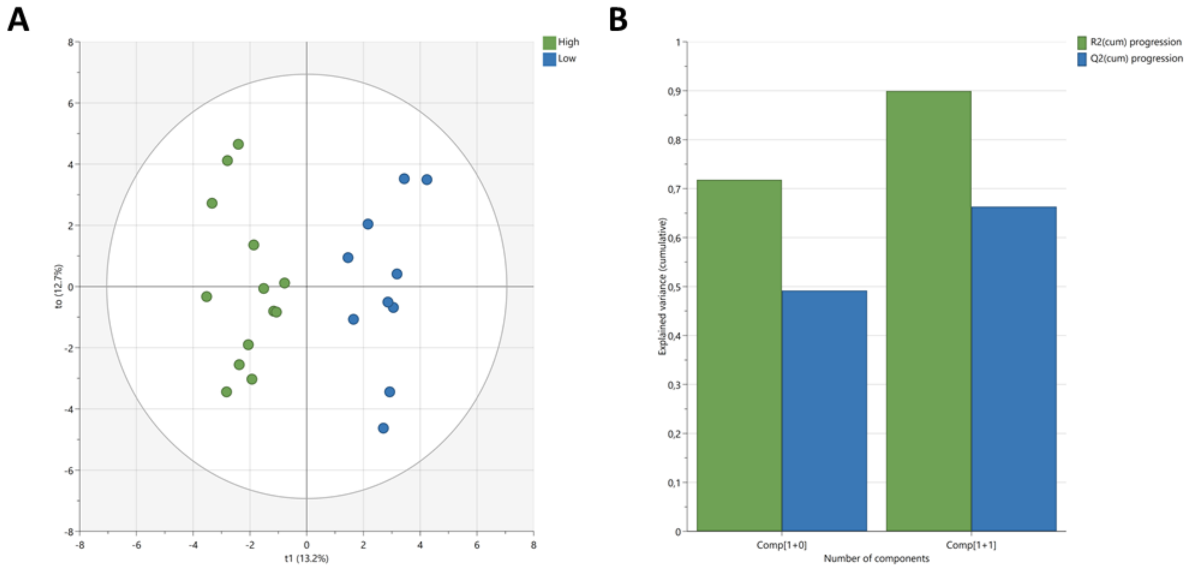

Figure 6 Model fit of the orthogonal partial least squares discriminant analysis (OPLS-DA) separating high and low post-exercise blood lactate concentration groups. A) Scores plot. B) Cumulative percent of the variation of the response explained by the model after the last component in the OPLS-DA.

Figure 7

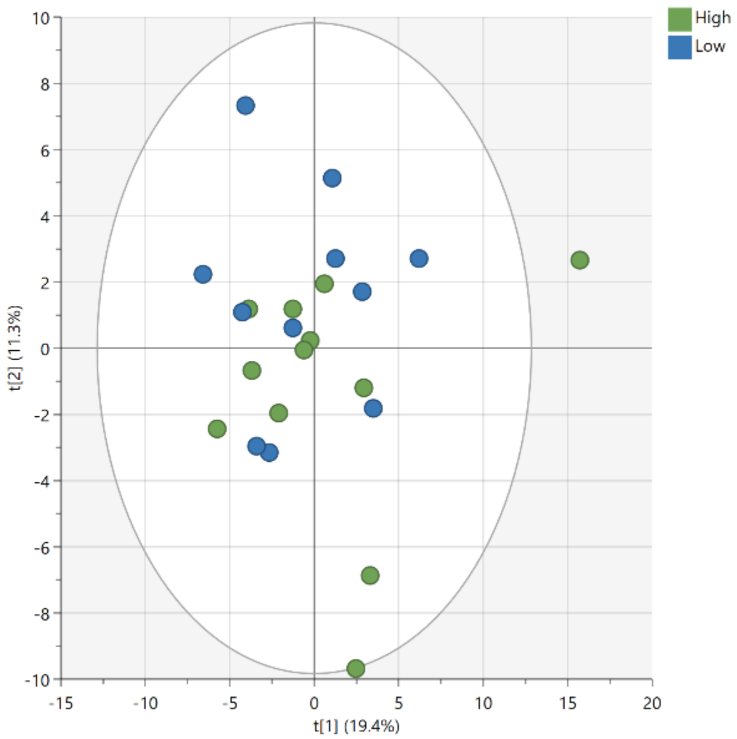

Figure 7 Principal component analysis showing higher- vs. lower-performing sprint skiers.

Figure 8

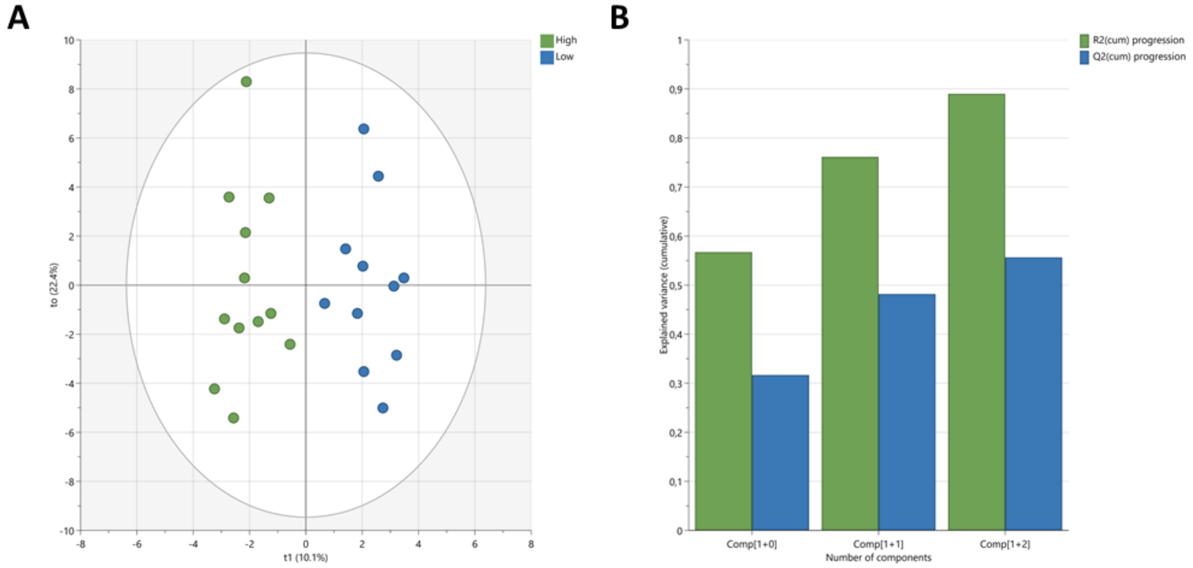

Figure 8 Model fit of the orthogonal partial least squares discriminant analysis (OPLS-DA) separating higher- and lower-performing sprint skiers. A) Scores plot. B) Cumulative percent of the variation of the response explained by the model after the last component in the OPLS-DA.

Figure 9

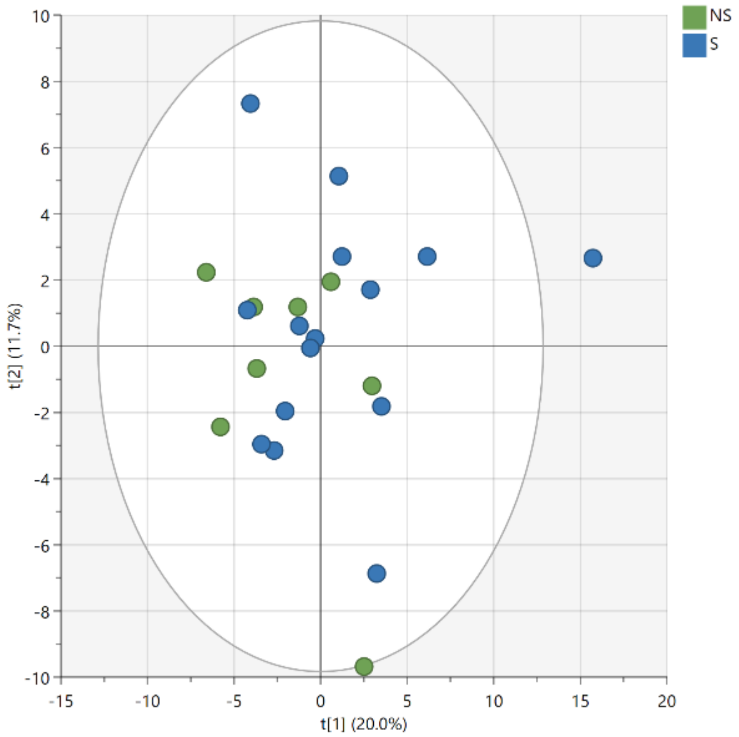

Figure 9 Principal component analysis showing the resting metabolic profiles of non-susceptible (NS) vs. susceptible (s) athletes.

Figure 10

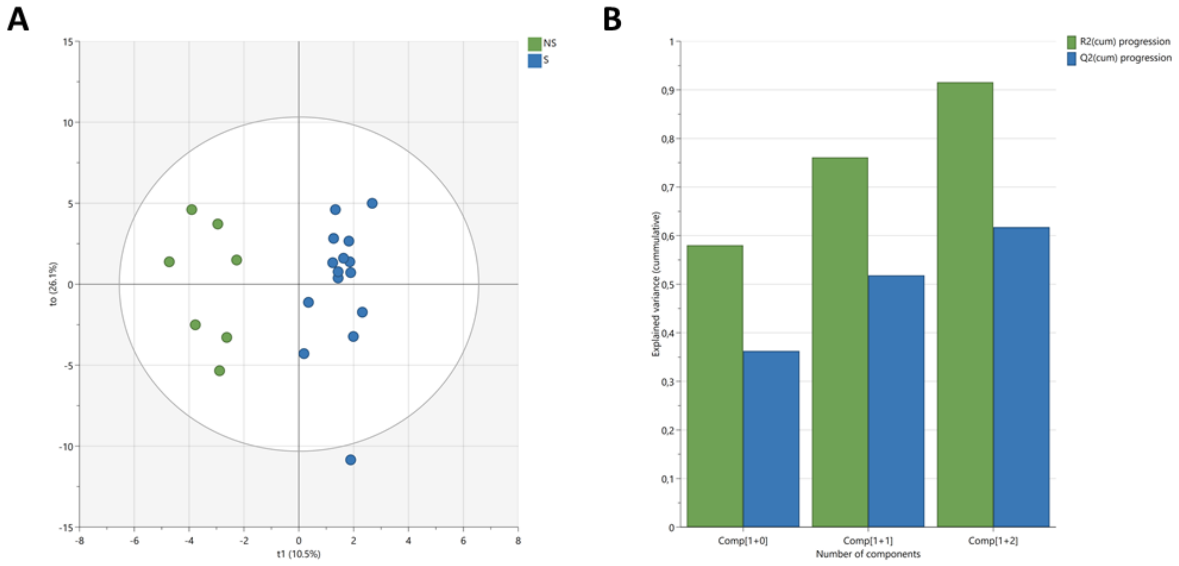

Figure 10 Model fit of the orthogonal partial least squares discriminant analysis (OPLS-DA) separating the resting metabolic profiles of non-susceptible (NS) and susceptible (S) athletes. A) Scores plot. B) Cumulative percent of the variation of the response explained by the model after the last component in the OPLS-DA.
